# Exploration and detection of potential regulatory variants in refractive error GWAS

**DOI:** 10.1038/srep33090

**Published:** 2016-09-08

**Authors:** Xuan Liao, ChangJun Lan, Dan Liao, Jing Tian, XiuQi Huang

**Affiliations:** 1 Department of Ophthalmology, Affiliated Hospital of North Sichuan Medical College, Nanchong 637007, Sichuan Province, China; 2 Department of Ophthalmology and Optometry, North Sichuan Medical College, Nanchong 637007, Sichuan Province, China

## Abstract

Refractive error (RE) is a complex multifactorial disease. Genome-wide association studies (GWAS) have provided significant insight into the genetic architecture and identified plenty of robust genetic variations or single nucleotide polymorphisms (SNPs) associated with complex disease. A major current challenge is to convert those resources into causal variants and target genes. We used RegulomeDB and HaploReg to annotate regulatory information onto associated SNPs derived from the two largest RE GWAS, and additional SNPs in linkage disequilibrium (LD) with GWAS significant SNPs. Overall 868 SNPs were investigated, out of which 662 returned RegulomeDB scores of 1 to 6. It was observed that 36 out of those SNPs show strong evidence of regulatory effects with a RegulomeDB score of 1, while only four of them were GWAS significant SNPs (*CD55*/rs1652333, *CNDP2*/rs12971120, *RDH5*/rs3138142 and rs3138144). The results encourage us to explore those putative pathogenic variants, both GWAS significant SNPs as well as the SNPs in LD, for future discernment of functional consequence. This study offers the attractive approach for prioritizing potential functional variants by combining ENCODE data and GWAS information, and provide further insights into the pathogenesis and mechanism and ultimately therapeutics.

Genome-wide association study (GWAS) approach has gained momentum nearly a decade ago in human genetics, and become a major strategy to examine the genetic basis of common complex diseases^1^. To date, nearly 1800 human GWAS have been successfully conducted to identify thousands of single nucleotide polymorphisms (SNPs) associated with many diseases or phenotypes, and documented in the GWAS catalog of the National Human Genome Research Institute (NHGRI)^2^. Although GWAS have offered remarkable insight into the genetic architecture, a major challenge in the interpretation of GWAS results is to find direct biological evidence that link the associated variants to the diseases or phenotypes. However, genetic association signals at any risk locus have become increasingly complex due to numerous index SNPs, and their respective proxy SNPs; the former revealed by GWAS significance (*P*-value) and the latter defined by correlation coefficient (*r*^2^) threshold of population-specific linkage disequilibrium (LD). Moreover, the vast majority of variants identified are generally of weak to modest effect sizes attributing to the common disease/common variant hypothesis. Alongside these, 90% or more of associated variants have been located outside protein-coding genes such as intergenic and intronic regions, implying the non-coding regions may be crucial to uncover the massive genetic information of human genome^3^.

The exploration for functional annotation of non-coding variants has been greatly facilitated by progress in genome projects, complemented by advances in bioinformatic resources. The Encyclopedia of DNA elements (ENCODE) and other projects can provide better interpretation of the non-coding sequences of the genome, as revealing that large tracts of important regulators of gene expression locate somewhere in the desert regions lacking coding genes and thereby biological functions as previously considered^4^. Researchers have utilized multiple molecular techniques to identify the functional elements like transcriptions factors, protein bounds and motifs, histone modifications sites, DNA methylation and DNase hypersensitivity sites^5^. It implies that the underlying mechanism linking these variants to the diseases or phenotype is regulatory rather than coding. On that basis, RegulomeDB^6^ and HaploReg^7^ databases integrating ENCODE and other data, were developed to enable the regulatory and epigenomic annotation onto any set of variants derived from GWAS studies or genomic sequencing. These novel tools are not only useful but essential to expanding understanding of introgenic and intergenic variants that may alter regulatory function, gene expression, and ultimately disease phenotypes. Also, these databases, when applied to complex diseases, provides a rich source of information that can be used to associate GWAS data with functional annotations in an increasingly context specific manner.

Refraction error (RE) is a complex multifactorial disease that tends to show merely moderate associations among a number of genes. Investigation of the regulatory activity of variants, in this regard, will contribute to our understanding of the association between the variants and disease. Myopia or nearsightedness is one of the major subtypes of spherical RE. To date, the two largest independent GWAS, for the first time yielding high statistical power, had remarkably achieved major progress in the field^8^,^9^. Both studies have not only unearthed more than 20 loci significantly associated with RE and myopia age at onset respectively, but also confirmed associations to the *RASGRF1* and *GJD2* loci previously reported in two RE GWAS in British^10^ and Dutch populations^11^. One of them, from the international Consortium for Refractive Error and Myopia (CREAM), independently identified 21 associated loci in its multiethnic panel^8^. Another GWAS, by the direct-to-consumer genotyping company 23andMe Inc., successfully identified a total of 22 risk loci in a European derived population^9^. These results are strikingly similar and could provide inspection and verification by each other^12^. In order to prioritize potential regulatory variants, we performing functional annotation of the original GWAS SNPs themselves and their many proxy SNPs using three web-based tools, namely SNAP (http://www.broad.mit.edu/mpg/snap)^13^, RegulomeDB (http://regulomedb.org)^6^ and HaploReg (http://compbio.mit.edu/HaploReg)^7^,^14^.

## Results

The SNAP searches yielded 846 and 122 proxy SNPs in LD with the 40 GWAS significant SNPs at *r*^2^ thresholds of 0.8 from 1000 Genomes and HapMap, respectively. After removing overlaps, a total of 868 proxy SNPs were available for analysis. By repeating this step with higher thresholds, the SNAP portal found 592 (*r*^2^ ≥ 0.9) and 198 (*r*^2^ = 1.0) proxy SNPs from both databases. It turned out that these higher *r*^2^ thresholds yielded less number of identified SNPs.

We then examined 868 SNPs for possible regulatory functions using RegulomeDB database. RegulomeDB scores of 1 (most likely to affect binding and expression of target gene) to 6 (lest likely) were assigned for each of 662 SNPs, remaining variants had no data available. It is noteworthy that lesser the scores, more likely it would be that variant lies within a potential functional region. Of these 662 SNPs, 61 possessed strong regulatory potential with the score ≤2. Furthermore, 36 SNPs demonstrated relatively more evidence with the score of 1, specifically including one with a score of 1a, 5 score of 1b, 5 score of 1d and 25 score of 1f. Detailed information about the potential regulatory SNPs is shown in Table 1. The scores for the potential regulatory SNPs are given in Table 2. Note that only 4 of the 36 variants were GWAS significant SNPs (rs3138142, rs3138144, rs1652333 and rs12971120), and the rest 32 SNPs were in linkage disequilibrium with the index SNPs identified in RE GWAS (*r*^2^ ≥ 0.80).

Total of 5 RE GWAS significant loci, including *MYO1D*, *TJP2*, *RDH5*, *CD55* and *CNDP2*, contained variable number of potential regulatory SNPs (RegulomeDB score = 1). For the *MYO1D* gene region, three potential regulatory SNPs of the 34 proxy SNPs investigated were in LD with the GWAS significant SNP *MYO1D*/rs17183295 that itself had minimal functional evidence (RegulomeDB score = 5). Analogous situation may be found in the *TJP2* region, 3 functional SNPs of 18 proxy SNPs were in LD with the GWAS significant *TJP2*/rs11145746 (RegulomeDB score = 6). Meanwhile, 2 proxies of the *RDH* gene in LD with the GWAS significant *RDH5*/rs3138142 and *RDH5*/rs3138144 were analyzed, 13 of 88 proxies with *CD55*/rs1652333, and 15 of 29 proxies with *CNDP2*/rs12971120 (RegulomeDB score = 1, each). Summary of LD between GWAS significant SNPs and 36 proxy SNPs with regulatory potential is given in Table 2. Interestingly, the GWAS significant SNP in the *RDH* gene showed complete LD with itself, but not with any other regulatory polymorphism.

The variant rs10512441 had the strongest evidence of regulatory potential with a score of 1a for either the 3 functional proxies on the *MYO1D* locus or the 36 functional proxies on those five risk loci. The top SNP is an intergenic variant lay between the *MYO1D* and *TMEM98* regions, 15 kb 5′ to the *TMEM98* gene transcription start site. According to HaploReg, the SNP locates within a DNase I hypersensitive region reported in about 22 different cell types, but histone modification data for H3K27ac, H3K9ac, H3K9me1 or H3K9me3 are unavailable. It is situated within the binding sites of 24 proteins including EP300, POLR2A, CTCF, TRIM28, FOXA2, JUND, GATA3, UBTF, SRF, TAF1, YY1, ZBTB7A, ZNF143, SP1, ARID3A, HDAC2, FOXA1, TBP, MYC, PML, PHF8, MAZ and KAP1, HDAC2. Moreover, the variant significantly alters HNF3beta, Foxa and Foxj2 transcription factor binding motifs. As with two other functional SNPs rs17183628 and rs17781142 in intronic locations, the strongest SNP rs10512441 is the expression quantitative trait loci (eQTL) affecting expression of *MYO1D* and *TMEM98*. Rs17183628 falls within protein binding of TEAD4 and mediates Zfp691, ZFP652, FXR, Pax4 and VDR binding motifs. Histone modification ChIP-seq peaks verify its presence to a transcriptionally regulatory locus in multiple cell lines. Rs17781142 has indications for different proteins binding of JUND, EP300, PML, POLR2A, MYC, GATA1, GATA2, TAF7, and so on. There are some overlap proteins binding sites between rs17781142 and rs10512441, such as EP300, POLR2A, JUND, MYC and PML. Also, the variant rs17781142 is connected with a DNase I hypersensitive site and histone modification marker.

All the 3 functional proxies in the *TJP2* gene region, including intergenic variant rs11145326 and intronic variants rs1538583 and rs11145488, are eQTLs for *TJP2.* Rs1538583 shows more evidence of regulatory potential with a score of 1b: maps to the binding sites (EBF1, PML and NFIC) and changes the motifs (POU3F2, Foxa, Pbx3, Pou1f1, Pou3f2 and STAT), as well as involves histone markers and DNase sites. Rs11145326 is associated with the proximal and distal transcriptional regulation of *TJP2* and *ENSG00000227410*, and lies 5′ to the *TJP2* transcription start site within binding sites of PAX6, SOX, etc. Rs11145488 significantly disrupts Nanog and Sox transcription factor binding sites and lies within a DNase I hypersensitive region. Histone marks spotted this variant in an active locus. However, protein binding sites containing this variant are yet to be determined.

The GWAS significant variant rs3138142, located in a synonymous coding area of *RDH5*, presents within protein binding site of POLR2A, as well as alters Nr2f2 and PLAG1 and RXRA motifs. Another reported significant variant rs3138144 locates in intron of *RDH5*, overlapping binding site of POLR2A, changing the binding affinity of BCL and NRSF. They all are associated with histone markers and DNase sites in a variety of cell types, and affect proximal transcriptional regulation of *RDH5.* The reported GWAS significant SNP rs1652333 in intergenic region with regulatory evidence is an eQTL for *DAF*. Similar to other functional proxy SNP in our study, rs1652333 significantly disrupt AP-4, E2A, Evi-1, Mef2, Myf and RP58 transcription factor binding sites. CHIP-seq data indicate that the variant maps to the binding sites of MEF-2, AP-4, MYF6, TCF21 and TFAP4 protein. There are other seven intergenic (rs1346720, rs7545125, rs12095015, rs2802236, rs1572275, rs2564974 and rs2564978) and five intronic (rs4844592, rs6700168, rs10864231, rs1507758 and rs1507760) polymorphisms in the *CD55* region with putative regulatory function. Particularly, the GWAS significant rs12971120 in intronic region is an eQTL for *CNDP2* but does not affect any known protein and motif. While 14 other SNPs in the same gene are eQTLs for *CNDP2* and map to few of proteins namely SIN3A, GATA2, TAL1, MYC and PAX5. Meanwhile, those SNPs change binding affinity of Pax5, TEAD1, EGR2, Sox13, Zic1, etc. Furthermore, 4 of 15 identified SNPs with regulatory evidence are situated in other regions than intron, including 5′UTR (rs3764509), non-synonymous coding area (rs2278161), synonymous coding area (rs2303463 and rs2278159).

## Discussion

The explosive growth number of robust and replicable genetic associations has increased the urgency and complexity of understanding the biological foundations underlying genetic signals for complex disease. As the majority of GWAS-identified variants map to non-coding sequences, their effects can be realized via gene expression regulation or transregulatory activity. Advancing from statistical associations to functional annotation has provided further insights into this field with a collection of genome database that will enable the biological interpretation of GWAS signals. These publicly available annotation resources are commendable for teasing out potential causal variants and candidate target genes, as well as their possible functional consequence^15^,^16^. The present study demonstrates the functional assignment of regulatory information onto RE-associated SNPs, and attempts to provide the reader with the attractive tools for predicting the regulatory potential of variants.

Of the total 35 GWAS-associated loci for RE, five (*MYO1D*, *TJP2*, *RDH5*, *CD55* and *CNDP2*) contained SNPs with functional evidences which were involved in transcriptional regulatory processes and enriched with eQTL in a tissue-specific manner. Remarkably, of the total 40 GWAS significant SNPs, only four SNPs (*CD55*/rs1652333, *CNDP2*/rs12971120, *RDH5*/rs3138144 and rs3138142) showed a putative functional role on the basis of RegulomeDB score 1f or 1d, respectively. According to the findings, it seems that the four GWAS significant SNPs not their proxy SNPs contribute to RE susceptibility through regulatory properties that impact translational efficiency and protein level; however this needs to be investigated experimentally. Dissimilarly, none of the SNPs in *MYO1D* and *TJP2* regions with strong evidence of regulatory function is GWAS significant SNP, i.e. *MYO1D*/rs17183295 and *TJP2*/rs11145746. The associated variants identified in GWAS may in fact only be linked to, rather than themselves be, the causal variants. In this regard, a clear distinction between the true and surrogate signal is difficult mainly due to linkage disequilibrium that permits multiple variants at the same phenotype-associated locus even if only one of them is causal. Furthermore, those proxy SNPs, mapping in not only 5′UTR and exonic regions but intergenic and intronic regions, suggested that regulatory elements throughout the human genomic regions and gene expression stages. It complicated the detection of the loci affected by particular regulatory elements and the analysis of the interconnection of various regulatory networks. As discussed in the present study, the identification of suitable functional variants may be especially effective in prioritizing efforts for these loci.

Thirteen putative regulatory SNPs in the *CD55* gene region are all eQTLs for *CD55* or decay-accelerating factor (*DAF*). *CD55/DAF*, a 70 kd phosphatidyl-inositol anchored glycoprotein, is a member of the cell membrane bound complement regulatory proteins that inhibit autologous complement cascade activation. *CD55/DAF* protects cells from complement-mediated damage by inhibiting the formation and accelerating the decay of C3/C5 convertases. It binds activated complement fragments C3b and C4b, thereby inhibiting amplification of the complement cascade on host cell membranes^17^. The membrane regulatory proteins may serve as an important mechanism of self protection and render autologous cells insensitive to the action of complement. Expression of the gene has been demonstrated in human eye tissue, including retinal RPE, photoreceptors and choroid^8^. *CD55/DAF* also is known to elevate cytosolic calcium ion concentration^8^,^18^. Over-representation of calcium ion gene has been shown in the experimental myopia model by genome-wide scleral miRNA and mRNA profiling^19^. Also, fifteen putative regulatory SNPs in the *CNDP2* gene region are all eQTLs for cytosolic nonspecific dipeptidase isoform 2 (*CNDP2* or *CN2*) named previously tissue carnosinase. The pathophysiological relevance of *CNDP2* is degradation of carnosine (β-alanyl-L-histidine), which is an important bioactive dipeptide^20^. *CNDP2* belongs to the family of M20 metallopeptidases, which play key roles in regulating cell matrix composition and have been implicated in normal development process and various disease pathogenesis^21^,^22^. Matrix metalloproteinases are essential for remodelling of the extracellular matrix (ECM) and growth of the eye. Some of metallopeptidases levels in the sclera or aqueous humor are known to be associated with axial length and refractive power^23^,^24^,^25^.

There have been only few SNPs on an overlap of GWAS loci and eQTL in the *MYO1D* gene region. Particularly, *MYO1D*/rs10512441 with a lower RegulomeDB score of 1a indicates a higher likelihood to affect binding and gene expression. *MYO1D* (myosin-1d), encoding a putative binder of calmodulin, is a monomeric actin-based motor found in a wide range of tissues, such as cornea, choroid, retina photoreceptors and retinal pigmented epithelium (RPE). *MYO1D* mediates calcium ion sensitivity to *KCNQ5* ion channels, which participates in the transport of potassium ions from retina to choroid and may contribute to voltage-gated potassium ion channels in the photoreceptors and retinal neurons associated with myopia^26^,^27^. Two SNPs in the *RDH5* region, both as genome-wide significant SNPs and putative regulatory SNPs, are eQTLs for retinol dehydrogenase-5 (*RDH5*). The contribution of *RDH5* has been demonstrated in the visual cycle. *RDH5* is one of the key factors in the regeneration of 11-cis retinal, the light sensitive component of photoreceptors in the RPE^28^. Mutations in *RDH5* have been linked with fundus albipunctatus, a rare form of congenital stationary night blindness (MIM 136880) associated with myopia^29^. *RDH5* also is involved in retinoic acid (RA) metabolic process to catalyze oxidation of retinol to retinaldehyde. RA is highly expressed in the choroid and may mediate the transfer of myopic signal from the retina to the sclera, which has been implicated in eye growth in form-deprived myopia and lens-induced myopia^30^,^31^,^32^. Three SNPs in the *TJP2* region are eQTLs for tight junction protein 2 (*TJP2)*, also known as zona occludens 2 (*ZO*-2). It belongs to the membrane associated guanylate kinase-like protein family^33^. *TJP2* also serves as scaffolds for signaling proteins and transcription factors that regulate vesicular traffic as well as cell proliferation and differentiation. *TJP2* has been linked with hearing loss, and its duplication and subsequent over-expression are found in adult-onset progressive nonsyndromic hearing loss^34^. However, *TJP2* has not yet a known role in vision development or the vision cycle, and would be worthy of further experimental investigation.

According to RegulomeDB and HaploReg, the binding of the paired box 6 (PAX6) and SRY-box (SOX7, 8, 15) is affected by *TJP2*/11145326 (score = 1f). *PAX6* (OMIM 607108) is a highly conserved member of a family of transcription factors containing the paired box and homeobox domain that binds DNA, regulates gene expression, and is closely involved in oculogenesis^35^. Astoundingly, we did not find the index SNPs or any proxy SNPs that have a strong regulatory potential in *PAX6* gene region, whereas our results suggested that this gene was affected by other SNPs in different genes with evidence of regulatory function. What’s more, *PAX6* has been vigorously studied in high myopia, and both functional and linkage evidence have suggested that *PAX6* plays a role in the control of eye globe growth. Animal studies showed a significant changes in *PAX6* expression level in form-deprivation myopia^36^. Also, *PAX6* gene dosage influencing normal eye development and overexpression can cause microphthalmia^37^. Mutations in *PAX6* are responsible for aniridia, presenile cataract, aniridia-related keratopathy, and foveal hypoplasia^38^. The genomewide linkage scan showed that *PAX6* underlies the highest point of the peak on 11p13 with LOD 6.1^39^. Meanwhile, it is noteworthy that another the highest LOD scores were on 3q26, where located *SOX2* gene, a member of the family of sex-determining region Y-box (*SOX*) transcription factor genes. Mutations of *SOX2* can result in anophthalmia or microphthalmia^40^,^41^. Thus, the potential functional link between *TJP2* and RE deserves further investigation.

Also, we identified a couple of possible functional polymorphisms located in *RHD5* (including intron rs3138144 and synonymous rs3138142), all falling within the protein binding of POLR2A. POLR2A (aka RPB1) is a subunit DNA-directed RNA polymerase II involved in RNA synthesis and a platform for modifications specifying the recruitment of factors that regulates transcription, mRNA processing, and chromatin remodelling^42^. POLR2A has a unique C-terminal domain, which has been linked to DNA interaction and histone displacement during elongation. Based on RegulomeDB and HaploReg, its binding is linked to a considerable amount of SNPs in various genes, e.g., *CD55*, *MYO1D*, *CNDP2*, and *RDH5*. Likewise, we found other common protein binding sites that appeared to be linked to the polymorphisms at different RE-associated loci. This process gave the results listed in Table 2. The results indicated that there was potential functional link among these genes through some common signal pathway involved in pathogenesis and biology of RE. Apart from that, certain loci contain significantly more polymorphisms that have been detected for the putative regulatory function than others. However, it doesn’t reach a decision that the amount of potential functional polymorphisms is responsible for the magnitude of risk loci on disease pathogenesis and progression.

These approaches complement statistical identification of a number of associated variants with further functional annotations and biological predictions, but the results of the current study should be addressed within the context of its limitations. Firstly, these databases provide information only for allowing us to examine nucleotide variations responsible for chromatin state, conservation and their effect on regulatory motifs. Therefore, they are not yet to explicitly uncover the roles of these variants involving some other regulatory mechanisms or pathways like RNA splicing and miRNA processing regulation. As a complex disease, RE depends on the interaction of multiple genetic and environmental factors, which is likely linked with epigenetic effects on chromation modification, histone modification and DNA methylation. Efforts should be made to find out in what way the environmental factors affects the regulatory elements. Furthermore, RegulomeDB system represents an early functional annotation of the genome, yet still leaving a certain amount of SNPs to be determined. A total of 206 (23.73%) SNPs of the 868 exhibited “No data” that made it difficult to establish their involvement in RE. It needs more functional SNPs validated to match annotations from specific tissues allowing for even more prediction of molecular and phenotypic outcomes. In spite of this, RegulomeDB and HaploReg have provided important insights on the impact of the genetic variants both coding and non-coding regions in genome. As additional functional data are collected from a variety of sources, there is every reason to believe that these limitations will be reduced.

In summary, these databases RegulomeDB and HaploReg based on the experimental or computational evidences, made it easy to map regulatory regions and derive a valid hypothesis as to its function. We therefore identified a few of potential regulatory SNPs and susceptible loci, as well as discovered several proteins interacting with each other. Also, these results suggested that it was important to scrutinize LD pattern of associated SNPs, which will contribute to understanding the relationships between the variants and diseases, and detecting true causal variants in genetic association studies. Beyond that, it may be beneficial to elucidating the common genetic mechanisms and pathways between complex diseases. In this scenario, those databases and approach have significant value in selecting potential functional variants on the regulatory region for future discernment of functional consequence and hence the biological basis of RE and other complex diseases.

## Methods

### Index SNPs selection

Genome-wide significant SNPs were selected initially as our index SNPs of interest from the above-mentioned GWAS, which were approved by Institutional Review Board and Medical Ethics Committee of each participating center. Included among these were the 21 SNPs in CREAM study and 22 SNPs in 23andMe study from 35 risk loci of RE and AAO of myopia, which contains overlapping genes (*PRSS56*, *BMP3*, *LAMA2*, *RDH5*, *TOX*, *ZIC2*, *GJD2*, *RASGRF1*) and non-overlapping genes (*CD55*, *CHRNG*, *CACNA1D*, *CHD7*, *ZMAT4*, *RORB*, *CYP26A1*, *BICC1*, *GRIA4*, *PCCA*, *MYO1D*, *KCNJ2*, *CNDP2*, *LRRC4C*, *RBFOX1*, *KCNQ5*, *SFRP1*, *SHISA6*, *TJP2*, *RGR*, *DLG2*, *ZBTB38*, *PDE11A*, *DLX1*, *KCNMA1*, *BMP4* and *PABPCP2* pseudogene) in both studies. After merging one identical SNP (rs524952) and removing two deficient SNPs (chr8:60178580, chr14:54413001, without dbSNP rs identifiers and data), a total of 40 genome-wide significant SNPs (*P* < 5 × 10E-8) were available for the present analysis (see Supplementary Table S1).

### Proxy SNPs identification

After selecting index SNPs for identifying potential regulatory functions, the SNAP web portal was accessed on 2 December 2015. SNAP contains information of proxy SNPs with different LD values, basing on two genome databases (HapMap and 1000 Genomes)^13^. To assess whether index SNPs are linked to any potential functional SNPs, we utilized the SNAP portal to identify all proxy SNPs in LD with published SNP above *r*^2^ threshold of 0.80 (*r*^2^ ≥ 0.80), using the CEU populations from both HapMap version 3 or 1000Genomes pilot 1 projects. Subsequently, proxy SNPs were queried in stronger LD (*r*^2^ ≥ 0.90 and *r*^2^ = 1.0, respectively) with the index SNPs to further understand the associated SNPs. The results for all index SNPs along with their respective proxy SNPs at each *r*^2^ threshold values from both genome databases are summarized in Supplementary Table S1.

### Functional SNPs prioritization

Following index SNPs selection and proxy SNPs identification at *r*^2^ threshold of 0.80, we firstly employed RegulomeDB to identify and compare potential regulatory variants. RegulomeDB presents a classification scheme based on strength of experimental evidences (ChIP-Seq, eQTL) or computational predictions (DNase footprinting, position weight matrices) where a variant located in functional region likely results in a functional consequence (see Table 3). The heuristic system adopts four categories with scores of 1–6 that indicate additional annotations from the most confident to the least confident. Category 1 is further divided into subcategories 1a to 1f, and a variant scored as 1a has the highest confidence on functionality. The scores for all these variants are shown in Supplementary Table S2. Simultaneously, we utilized HaploReg to further annotate those filtered variants and facilitated to discover their potential causal link with the disease pathogenesis. Besides the above, this tool labels SNPs using evolutionary conserved genome sequences (GERP and SiPhy scores), epigenomic alterations (ChromHMM, histone modification ChIP-seq) and enrichment analysis. HaploReg further provides the functional prediction of potential causal variants and candidate risk loci by systematic mining of comparative, regulatory and epigenomic annotations.

## Additional Information

**How to cite this article**: Liao, X. *et al.* Exploration and detection of potential regulatory variants in refractive error GWAS. *Sci. Rep.* **6**, 33090; doi: 10.1038/srep33090 (2016).

## Supplementary Material

Supplementary Information

## Figures and Tables

**Table 1:** Annotation of potential regulatory SNPs in RegulomeDB Category of 1.

Chromosome: Location	RefSNP^1^	Allele	Gene	Position	Histone marked^2^	DNase	eQTL	Motifs altered^3^	Protein bound^3^
1:207387765	rs1346720	T/C	*CD55*	intergenic 69 kb 3′ of *C4BPA*	−	−	+	−	SETDB1*
1:207424726	rs7545125	A/G	*CD55*	intergenic 70 kb 5′ of *CD55*	+	−	+	−; DIx2, Foxo, Ik-2, NF-AT, NF-AT1, YY1	CEBPB*
1:207456473	rs12095015	T/C	*CD55*	intergenic 38 kb 5′ of *CD55*	+	+	+	MGA; Foxp1, Irf, SRF, Zec	CDX2, JUN; CJUN
1:207470459	**rs1652333**	G/A	*CD55*	intergenic 24 kb 5′ of *CD55*	+	−	+	−; Ap-4, E2A, Evi-1, Mef2, Myf, RP58	MEF-2, TCF21, AP-4, TFAP4, MYF6
1:207472188	rs2802236	T/C	*CD55*	intergenic 23 kb 5′ of *CD55*	+	+	+	HOXA5(Hox-1.3); Eomes, Hoxa5, Pax5, Pax6, Zfp187	FOXA1, ESR1, EP300, GATA3*; ERALPHA-A
1:207478979	rs1572275	C/A	*CD55*	intergenic 16 kb 5′ of *CD55*	+	+	+	−; AIRE, Pou5f1	SMARCC1, RAD21*, POLR2A, CEBPB*, TAF1*, NFYB, TBP*, CHD2, TFAP2A
1:207483444	rs2564974	G/A	*CD55*	intergenic 11 kb 5′ of *CD55*	+	+	+	Irx-3, Irx3, Irx4, Irx6, TFAP4; CDP, Irx, Myb	STAT1, SMARCC1, TCF7L2, SP1, EP300, TCF12, MYC, ELF1*, RCOR1, MEF2A, TEAD4, SPI1, BHLHE40, JUND*, GATA2*, TAL1*, JUN, USF1, MAX*, GATA1, POLR2A, CBX3, SIRT6*; PU1*, POL2*, BAF155*, CJUN*, CMYC*, INI1*
1:207494415	rs2564978	T/C	*CD55*	intergenic 399 bp 5′ of *CD55*	+	+	+	FXR/RXR-alpha*	E2F1, MTA3, POLR2A*, SPI1, ELF1*, TAF1, CEBPB*, TBL1XR1, EP300*, TBP*, FOS, TRIM28, MYC*, STAT3*, KDM5B, ZNF143, HDAC1, MAX*, SP4, CDX2, MXI1, GATA1, STAT1, CTCF*, JUNB, PHF8, RAD21*, BACH1, TCF12, GABPB1, ZKSCAN1, IRF1, SIN3A, SETDB1, NFKB1*, HNF4A; PU1, GTF2F1, POL2S2, SMC3, CEBPB
1:207501210	rs4844592	T/A	*CD55*	intronic	+	+	+	Srf, SPI1, SPIC; HDAC2	POLR2A*
1:207502533	rs6700168	A/C	*CD55*	intronic	+	+	+	IRC900814	POLR2A*, NFYA, CHD2, MAX, RCOR1, SMC3, ZKSCAN1, ZNF143; POL24H8, GTF2F1, POL2B, POL2S2
1:207506328	rs10864231	T/G	*CD55*	intronic	−	+	+	−; PL2F, Pax5	POLR2A*, TAF1; POL24H8
1:207507480	rs1507758	G/C	*CD55*	intronic	+	+	+	−; Foxp1	POLR2A*, SETDB1*
1:207509364	rs1507760	C/T	*CD55*	intronic	+	+	+	−; NK-kappaB	POLR2A*, EBF1, CHD1, EP300, CTCF*, GATA1, RAD21*, NFKB1*, TBP, SP1, YY1, TBL1XR1, CHD2, RUNX3, BHLHE40, MXI1; POL24H8, POL2B
9:71733141	rs11145326	C/G	*TJP2*	intergenic 3.1kb 5′ of *TJP2*	−	−	+	−; MSX2, Pax6, STAT, Sox	PAX6, SOX15, SOX7, SOX8
9:71770938	rs11145488	G/A	*TJP2*	intronic	+	+	+	−; Nanog, Sox	
9:71791546	rs1538583	A/G	*TJP2*	intronic	+	+	+	POU3F2; Foxa, Pbx3, Pou1f1, Pou3f2, STAT	EBF1*, PML, NFIC
12:56114768	**rs3138144**	G/C	*RDH5*	intronic	+	+	+	−; BCL, NRSF	MYC, CEBPB, POLR2A, EP300, RUNX3, ZBTB7A, RXRA, MAZ, SMC3, CTCF*, E2F1
12:56115584	**rs3138142**	C/T	*RDH5*	exon, synonymous-coding	+	+	+	Nr2f2; PLAG1*, RXRA*	POLR2A
17:31184630	rs17183628	T/C	*MYO1D*	intronic	+	−	+	Zfp691, ZFP652; FXR, Pax4, VDR	TEAD4
17:31187215	rs17781142	G/C	*MYO1D*	intronic	+	+	+	−; LBP-1	JUN, PML, GATA2*, POLR2A, MAX, RCOR1, CEBPB, STAT2, GATA1*, STAT5A, EBF1*, TAF7, JUND, EP300, CCNT2*, MAZ, IRF1, NFKB1, EBF1, TBL1XR1, MYC*
17:31239644	rs10512441	C/T	*MYO1D*	intergenic 15 kb 5′ of *TMEM98*	−	+	+	HNF3beta; Foxa, Foxj2	EP300*, POLR2A*, CTCF*, TRIM28, FOXA2*, JUND, GATA3*, UBTF, SRF*, TAF1, YY1, ZBTB7A, ZNF143, SP1, ARID3A, HDAC2, FOXA1*, TBP*, MYC, PML, PHF8, MAZ; KAP1*, HDAC2*
18:72154930	rs747176	G/A	*CNDP2*	intergenic 8.6 kb 5′ of *CNDP2*	+	−	+	−; Mrg, Pax5, Pbx3, TAL1, Tgif1	
18:72167123	rs3764509	C/G	*CNDP2*	5′ UTR	+	+	+	−; Pbx3	POU2F2, SIN3A*, STAT3*, STAT1*, TAF1*, USF1*, TBP, USF2, SAP30, RUNX3, BHLHE40, CTCF, E2F6*, YY1*, CEBPB*; MAX, POLR2A, POL24H8, SIN3AK20, MYC, NFKB, MXI1, ETS1
18:72167801	rs12605820	G/A	*CNDP2*	intronic	+	+	+	Sfpi1; Foxo, HP1, Mef2, TCF12	
18:72168485	rs8084410	T/C	*CNDP2*	intronic	+	+	+	TEF*, TEAD1, TEAD4, TEAD3; DMRT4, RFX5, TCF12	SIN3A
18:72168607	rs2303463	G/A	*CNDP2*	exon, synonymous-coding	+	+	+	EGR2, EGR3	
18:72170298	rs4891557	C/T	*CNDP2*	intronic	+	−	+	ESR1, EWSR1-FLI1; ERalpha-a, EWSR1-FLI1, Irf, RXRA, VDR	
18:72170396	rs4891559	G/A	*CNDP2*	intronic	+	−	+	−; Bbx, Foxj2	
18:72174022	**rs12971120**	A/G	*CNDP2*	intronic	+	+	+		
18:72174979	rs3829640	A/G	*CNDP2*	intronic	+	+	+	−; Bcl6b	
18:72176082	rs2278161	T/C	*CNDP2*	exon, nonsynonymous- coding	+	+	+	−; BDP1	GATA2*, TAL1*, MYC
18:72177231	rs11151960	G/A	*CNDP2*	intronic	+	+	+	Sox13, Zic1, Zic2; E2F, Tel2	PAX5
18:72178160	rs2278159	T/C	*CNDP2*	exon, synonymous-coding	+	+	+	−; Maf	
18:72178299	rs2278158	G/T	*CNDP2*	intronic	+	+	+	−; Ets, TATA	
18:72179578	rs734559	G/A	*CNDP2*	intronic	+	+	+	Lyf-1, PTF1-beta; CEBPB, HMG-1Y, Ik-1, IK-2, NF-AT, NF-AT1, PTF1-beta, Pou5f1	
18:72182964	rs3794950	G/A	*CNDP2*	intronic	+	+	+	−; Jundm2	

RefSNP ID = reference SNP identification number in NCBI reference assembly, eQTL = expression quantitative trait loci, DNase = deoxyribonuclease.

^1^GWAS significant SNPs are bolded.

^2^Promoter and Enhancer histone marks are included.

^3^Data of motif altered and protein bound are separated by semicolons: before from RegulomeDB and after from Haploreg database. The repeated sites in both databases are marked with an asterisk.

**Table 2:** LD for GWAS significant SNPs with functional proxies.

GWAS significant SNP	Functional Proxy SNPs^#^	*r*^2^ threshold	RegulomeDB Score*
*MYO1D*/rs17183295	rs10512441	0.9	1a
	rs17183628	0.9	1d
	rs17781142	0.9	1f
*TJP2*/rs11145746	rs1538583	1.0	1b
	rs11145326	0.8	1f
	rs11145488	1.0	1f
*BLOC1S1* & *RDH5*/rs3138142	rs3138142	1.0	1d
*BLOC1S1* & *RDH5*/rs3138144	rs3138144	1.0	1f
*C4BPAP2/CD55*/rs1652333	rs1652333	1.0	1f
	rs2802236	1.0	1b
	rs2564974	1.0	1b
	rs2564978	1.0	1b
	rs12095015	1.0	1d
	rs6700168	1.0	1d
	rs1346720	0.8	1f
	rs7545125	1.0	1f
	rs1572275	1.0	1f
	rs4844592	1.0	1f
	rs10864231	1.0	1f
	rs1507758	1.0	1f
	rs1507760	1.0	1f
*CNDP2*/rs12971120	rs12971120	1.0	1f
	rs8084410	0.9	1b
	rs11151960	0.8	1d
	rs747176	0.8	1f
	rs3764509	0.9	1f
	rs12605820	0.8	1f
	rs2303463	0.8	1f
	rs4891557	0.8	1f
	rs4891559	0.9	1f
	rs3829640	1.0	1f
	rs2278161	1.0	1f
	rs2278159	0.8	1f
	rs2278158	0.8	1f
	rs734559	1.0	1f
	rs3794950	0.8	1f

^#^GWAS significant SNPs with regulatory potential are bolded, *RegulomeDB score are confined to the range 1a–f.

**Table 3:** Distribution of RegulomeDB variants categories^6^.

Category	Description	SNPs Number
Likely to affect binding and linked to expression of a gene target		
1a	eQTL + TF binding + matched TF motif + matched DNase footprint + DNase peak	1
1b	eQTL + TF binding + any motif + DNase footprint + DNase peak	5
1c	eQTL + TF binding + matched TF motif + DNase peak	0
1d	eQTL + TF binding + any motif + DNase peak	5
1e	eQTL + TF binding + matched TF motif	0
1f	eQTL + TF binding/DNase peak	25
Likely to affect binding		
2a	TF binding + matched TF motif + matched DNase footprint + DNase peak	1
2b	TF binding + any motif + DNase footprint + DNase peak	24
2c	TF binding + matched TF motif + DNase peak	0
Less likely to affect binding		
3a	TF binding + any motif + DNase peak	48
3b	TF binding + matched TF motif	1
Minimal binding evidence		
4	TF binding + DNase peak	85
5	TF binding or DNase peak	241
6	Motif hit	226
No data^#^		206

eQTL = expression quantitative trait loci, TF = transcription factor, DNase = deoxyribonuclease, ^#^No data is available in RegulomeDB scoring scheme.
