# Supplementary material for: Exploration and detection of potential regulatory variants in refractive error GWAS
Source: Sci Rep. 2016 Sep 8;6:33090. doi: 10.1038/srep33090 (PMC5015044; doi:10.1038/srep33090)
Supplement: Supplementary Information [file srep33090-s1.pdf]

Supplementary Information:

Exploration and detection of potential regulatory variants in refractive error GWAS

Xuan Liao, ChangJun Lan, Dan Liao, Jing Tian, XiuQi Huang

Supplementary Table S1. Index SNPs from published GWAS and Proxy SNPs from SNAP search in LD at tested r^2 thresholds.

Index SNP (Genes/Loci)*	Proxy SNP		
	$r^2 \geq 0.8$	$r^2 \geq 0.9$	$r^2 = 1.0$
**rs1652333**	rs1652333	rs1652333	rs1652333
C4BPAP2 (pseudogene)/CD55	rs2802233	rs2802233	rs2802233
	rs2802234	rs2802234	rs2802234
	rs2802236	rs2802236	rs2802236
	rs2782845	rs2782845	rs2782845
	rs971318	rs971318	rs971318
	rs971317	rs971317	rs971317
	rs2802238	rs2802238	rs2802238
	rs1572275	rs1572275	rs1572275
	rs2802217	rs2802217	rs2802217
	rs2564974	rs2564974	rs2564974
	rs12095015	rs12095015	rs12095015
	rs2017760	rs2017760	rs2017760
	rs1858001	rs1858001	rs1858001
	rs2914937	rs2914937	rs2914937
	rs891378	rs891378	rs891378
	rs891376	rs891376	rs891376
	rs10864181	rs10864181	rs10864181
	rs2564978	rs2564978	rs2564978
	rs10864180	rs10864180	rs10864180
	rs10864178	rs10864178	rs10864178
	rs2782828	rs2782828	rs2782828
	rs6703857	rs6703857	rs6703857
	rs6540896	rs6540896	rs6540896
	rs4844591	rs4844591	rs4844591
	rs4844592	rs4844592	rs4844592
	rs6700079	rs6700079	rs6700079
	rs6702997	rs6702997	rs6702997
	rs6703002	rs6703002	rs6703002
	rs6700168	rs6700168	rs6700168
	rs7548463	rs7548463	rs7548463
	rs925130	rs925130	rs925130
	rs925131	rs925131	rs925131
	rs2184476	rs2184476	rs2184476
	rs6662070	rs6662070	rs6662070
	rs10864231	rs10864231	rs10864231
	rs11120733	rs11120733	rs11120733
	rs1507758	rs1507758	rs1507758

rs10864175	rs10864175	rs10864175
rs7555030	rs7555030	rs7555030
rs7512422	rs7512422	rs7512422
rs1507760	rs1507760	rs1507760
rs1507761	rs1507761	rs1507761
rs113231892	rs113231892	rs113231892
rs1835307	rs1835307	rs1835307
rs7543250	rs7543250	rs7543250
rs7545125	rs7545125	rs7545125
rs112172464	rs112172464	rs112172464
rs111911746	rs111911746	rs111911746
rs877049	rs877049	rs877049
rs112720427	rs112720427	rs112720427
rs10864167	rs10864167	rs10864167
rs113896552	rs113896552	rs113896552
rs112511970	rs112511970	rs112511970
rs1429906	rs1429906	rs1429906
rs116218947	rs116218947	rs116218947
rs6664815	rs6664815	rs6664815
rs79515725	rs79515725	rs79515725
rs113849985	rs113849985	rs113849985
rs7522701	rs7522701	rs7522701
rs77542432	rs77542432	rs10746462
rs6703860	rs6703860	rs10746463
rs6681853	rs6681853	
rs11120586	rs11120586	
rs1507757	rs1507757	
rs12133548	rs12133548	
rs1507759	rs1507759	
rs113742197	rs113742197	
rs7533825	rs7533825	
rs1864370	rs1864370	
rs1354942	rs1354942	
rs10746462	rs10746462	
rs2564975	rs2564975	
rs4611012	rs4611012	
rs10864156	rs10864156	
rs6671700	rs10746463	
rs6540888	rs2135923	
rs1367067		
rs59036171		
rs1579484		

	rs11120688		
	rs1656404		
	rs2853443		
	rs2573206		
	rs733603		
	rs10746463		
	rs2135923		
	rs1346720		
**rs1656404**	rs1656404	rs1656404	rs1656404
ECEL1/PRSS56	rs2853443	rs2853443	
**rs1881492**	rs1881492	rs1881492	rs1881492
CHRNG	rs2573206	rs2573206	
	rs733603		
**rs14165**	rs14165	rs14165	rs14165
CACNA1D/CHDH	rs920252		
	rs893362		
	rs4687587		
	rs4687586		
	rs12676		
**rs1960445**	rs1960445	rs1960445	rs1960445
C4orf22&LOC105377305/BMP3	rs4458448	rs4458448	rs4458448
	rs963857	rs963857	rs963857
	rs10049776	rs10049776	rs10049776
	rs9307776	rs9307776	
	rs17005016	rs17005016	
	rs2868079	rs2868079	
	rs10004901	rs10004901	
	rs11099457		
	rs4693773		
	rs10003846		
	rs4693767		
	rs6814223		
**rs12205363**	rs12205363	rs12205363	rs12205363
LAMA2			
**rs4237036**	rs4237036	rs4237036	rs4237036
CHD7	rs13280978	rs13280978	rs13280978

	rs35168272	rs35168272	rs35168272
	rs4738826	rs4738826	rs4738826
	rs28609390	rs28609390	rs28609390
	rs1483208	rs1483208	rs1483208
	rs876957	rs876957	rs876957
	rs28710955	rs28710955	rs28710955
	rs4738829	rs4738829	
	rs4738828	rs4738828	
	rs7011801	rs7011801	
	rs10106752	rs10106752	
	rs1017862	rs1017862	
	rs1017861	rs1017861	
	rs10283352	rs10283352	
	rs13264416		
	rs1473446		
	rs10104525		
	rs35331770		
	rs4738816		
	rs28600309		
	rs4738814		
**rs7837791**	rs7837791	rs7837791	rs7837791
TOX& LOC100505501/	rs7832404	rs7832404	
RNA5SP267 (pseudogene)	rs7812962		
**rs7829127**	rs7829127	rs7829127	rs7829127
ZMAT4	rs16890057	rs16890057	rs16890057
	rs869422	rs869422	rs869422
	rs16890054	rs16890054	rs4736884
	rs11776583	rs11776583	
	rs2137277	rs2137277	
	rs2137278	rs2137278	
	rs72644322	rs72644322	
	rs72641600	rs72641600	
	rs35015019	rs35015019	
	rs4736884	rs4736884	
	rs72632903	rs72632903	
	rs7005382	rs7005382	
	rs6994354		
	rs4736886		
**rs7042950**	rs7042950	rs7042950	rs7042950

RORB	rs13289812	rs13289812	rs13289812
	rs10869416	rs10869416	
	rs10869417	rs10869417	
	rs10869418		
	rs13283886		
	rs117588997		
	rs7867494		
	rs7024262		
	rs10869412		
**rs10882165**	rs10882165	rs10882165	rs10882165
CYP26A1&NIP7P1 (pseudogene)/Exon	rs56871408	rs56871408	rs56871408
	rs11187304	rs11187304	
	rs6583867	rs6583867	
	rs10882169	rs10882169	
	rs7078201	rs7078201	
	rs7078310	rs7078310	
	rs7916761	rs7916761	
	rs12357177	rs12357177	
	rs11187313	rs11187313	
	rs11187314	rs11187314	
	rs7916838	rs7916838	
	rs7917125	rs7917125	
	rs4918865	rs4918865	
	rs10882172	rs10882172	
	rs7919934	rs7919934	
	rs7903804	rs7903804	
	rs17108260	rs17108260	
	rs17382981	rs17382981	
	rs72815184	rs72815184	
	rs72815186	rs72815186	
	rs7894625	rs7894625	
	rs117531235		
	rs12360392		
	rs12358810		
	rs10882163		
	rs11187298		
	rs10882157		
	rs72815193		
	rs55744367		
	rs57346439		
	rs7920484		

**rs7084402**	rs7084402	rs7084402	rs7084402
LOC105378316/ BICC1	rs12250013	rs12250013	rs12250013
	rs12259315	rs12259315	rs12259315
	rs12257983	rs12257983	rs12257983
	rs1863665	rs1863665	rs1863665
	rs1863664	rs1863664	rs1863664
	rs1346301	rs1346301	rs1346301
	rs1427204	rs1427204	rs1427204
	rs1427202	rs1427202	rs1427202
	rs717452	rs717452	rs717452
	rs4113952	rs4113952	rs4113952
	rs6481400	rs6481400	rs6481400
	rs6481401	rs6481401	rs6481401
	rs6481402	rs6481402	rs6481402
	rs7917717	rs7917717	rs7917717
	rs11006169	rs11006169	rs11006169
	rs34774248	rs34774248	rs34774248
	rs36097682	rs36097682	rs36097682
	rs1427201	rs1427201	rs1427201
	rs7910442	rs7910442	rs7910442
	rs7095501	rs7095501	rs7095501
	rs7073504	rs7073504	rs7073504
	rs7074778	rs7074778	rs7074778
	rs28426375	rs28426375	rs28426375
	rs1346302	rs1346302	
	rs7921207	rs7921207	
	rs7091440	rs7091440	
	rs7087998	rs7087998	
	rs11817440	rs11817440	
	rs7100474	rs7100474	
	rs7079648	rs7079648	
	rs7078900	rs7078900	
	rs10763551	rs10763551	
	rs10763552	rs10763552	
	rs10826198	rs10826198	
	rs10826199	rs10826199	
	rs7897298	rs7897298	
	rs1427200	rs1427200	
	rs1427199	rs1427199	
	rs1427198	rs1427198	
	rs1427197	rs1427197	

rs1896243	rs1896243
rs11006170	rs11006170
rs7094882	rs7094882
rs7098326	rs7098326
rs7097576	rs7097576
rs7098606	rs7098606
rs2170779	rs2170779
rs1365741	rs1365741
rs1365740	rs1365740
rs12242287	rs12242287
rs11006171	rs11006171
rs1427196	rs1427196
rs1649034	rs1649034
rs1649033	rs1649033
rs1649032	rs1649032
rs1649030	rs1649030
rs1658496	rs1658496
rs1658495	rs1658495
rs1658494	rs1658494
rs1649028	rs1649028
rs1649027	rs1649027
rs1658493	rs1658493
rs1658491	rs1658491
rs1649026	rs1649026
rs1649025	rs1649025
rs1658490	rs1658490
rs3001714	rs3001714
rs1649023	rs1649023
rs1649022	rs1649022
rs1658489	rs1658489
rs6481404	rs6481404
rs2577393	rs2577393
rs1658486	rs1658486
rs1649019	rs1649019
rs1649018	rs1649018
rs1658485	rs1658485
rs1658483	rs1658483
rs1649015	rs1649015
rs1658481	rs1658481
rs1658480	rs1658480
rs1649092	rs1649092
rs1658479	rs1658479

rs1649091	rs1649091
rs1658478	rs1658478
rs1658477	rs1658477
rs1427224	rs1427224
rs1427223	rs1427223
rs1427222	rs1427222
rs1593678	rs1593678
rs1593677	rs1593677
rs1593674	rs1593674
rs1649085	rs1649085
rs1649084	rs1649084
rs1658470	rs1658470
rs1658469	rs1658469
rs1649082	rs1649082
rs1649081	rs1649081
rs1658468	rs1658468
rs1649080	rs1649080
rs1649077	rs1649077
rs1649076	rs1649076
rs1593672	rs1593672
rs1626532	rs1626532
rs1658467	rs1658467
rs1649074	rs1649074
rs1582827	rs1582827
rs12259990	rs12259990
rs12261597	rs12261597
rs1649072	rs1649072
rs1658463	rs1658463
rs1658462	rs1658462
rs1303970	rs1303970
rs1303968	rs1303968
rs1658460	rs1658460
rs1658459	rs1658459
rs7087528	rs7087528
rs1658458	rs1658458
rs1619284	rs1619284
rs1658457	rs1658457
rs1649069	rs1649069
rs1649066	rs1649066
rs1649065	rs1649065
rs1658454	rs1658454
rs1614258	rs1614258

rs1649063	rs1649063
rs1649062	rs1649062
rs1649060	rs1649060
rs1658452	rs1658452
rs1658451	rs1658451
rs1649059	rs1649059
rs12569559	rs12569559
rs1658447	rs1658447
rs1658446	rs1658446
rs1620186	rs1620186
rs2114564	rs2114564
rs2438182	rs2438182
rs1820542	rs1820542
rs7099881	rs7099881
rs1658443	rs1658443
rs1649058	rs1649058
rs1649057	rs1649057
rs1658442	rs1658442
rs1649055	rs1649055
rs1623568	rs1623568
rs1621811	rs1621811
rs1658440	rs1658440
rs1649049	rs1649049
rs1649048	rs1649048
rs1658439	rs1658439
rs1649046	rs1649046
rs1658438	rs1658438
rs1658436	rs1658436
rs1649045	rs1649045
rs1658435	rs1658435
rs1621227	rs1621227
rs1620404	rs1620404
rs1626606	rs1626606
rs1649043	rs1649043
rs1649042	rs1649042
rs1658434	rs1658434
rs1649041	rs1649041
rs11815274	rs11815274
rs1658432	rs1658432
rs3001717	rs3001717
rs2917949	rs2917949
rs2577392	rs2577392

rs2842083	rs2842083
rs1649039	rs1649039
rs1658429	rs1658429
rs1649038	rs1649038
rs7921371	rs7921371
rs1649037	rs1649037
rs1658427	rs1658427
rs1658425	rs1658425
rs1658424	rs1658424
rs1658423	rs1658423
rs1658422	rs1658422
rs1649036	rs1649036
rs1658421	rs1658421
rs1649035	rs1649035
rs1658419	rs1658419
rs1658418	rs1658418
rs2393448	rs2393448
rs717453	rs717453
rs10763553	rs10763553
rs1896246	rs1896246
rs12264028	rs12264028
rs12248958	rs12248958
rs1427195	rs1427195
rs1658492	rs1658492
rs1632584	rs1632584
rs1593675	rs1593675
rs1658456	rs1658456
rs1649068	rs1649068
rs4141671	rs4141671
rs4948523	rs4948523
rs10763556	rs10763556
rs10763558	rs10763558
rs2393449	rs2393449
rs7474570	rs7474570
rs12263522	rs12263522
rs11006192	rs11006192
rs10763559	rs10763559
rs10740731	rs10740731
rs4948524	rs4948524
rs10740732	rs10740732
rs10826202	rs10826202
rs10763564	rs10763564

	rs893370	rs893370	
	rs1649021	rs1649021	
	rs1649089	rs1649089	
	rs1658471	rs1658471	
	rs1593673	rs1593673	
	rs1579394	rs1579394	
	rs1649067	rs1649067	
	rs1658455	rs1658455	
	rs1658453	rs1658453	
	rs1158212	rs1158212	
	rs1649051	rs1649051	
	rs1658441	rs1658441	
	rs1658428	rs1658428	
	rs1658426	rs1658426	
	rs1896244	rs1896244	
	rs7098329	rs7098329	
	rs1649031	rs1649031	
	rs7914888	rs7914888	
	rs3001715	rs3001715	
	rs1346300	rs1346300	
	rs1649017	rs1649017	
	rs1649016	rs1649016	
	rs1658484	rs1658484	
	rs1649079	rs1649079	
	rs1649078	rs1649078	
	rs1582826	rs1582826	
	rs1593670	rs1593670	
	rs1649044	rs1649044	
	rs1658417	rs1658417	
	rs10763557	rs10763557	
	rs4245599	rs4245599	
	rs1593671		
	rs1896245		
	rs4948525		
	rs7099496		
	rs2033149		
**rs11601239**	rs11601239	rs11601239	rs11601239
GRIA4	rs7106983	rs7106983	rs7106983
	rs11226825	rs11226825	rs11226825
	rs1838668	rs1838668	rs1838668
	rs994575	rs994575	rs994575

rs12790329	rs12790329	rs12790329
rs11226827	rs11226827	rs11226827
rs10791767	rs10791767	rs10791767
rs12417624	rs12417624	rs12417624
rs7926520	rs7926520	rs7926520
rs7931588	rs7931588	rs7931588
rs1938963	rs1938963	rs1938963
rs1954762	rs1954762	rs1954762
rs1445607	rs1445607	
rs10895857	rs10895857	
rs10895856	rs10895856	
rs977516	rs977516	
rs10895860	rs10895860	
rs1445618	rs1445618	
rs61900312	rs61900312	
rs1822993	rs1822993	
rs11226828	rs11226828	
rs1445619	rs1445619	
rs10895864	rs10895864	
rs1373926	rs1373926	
rs638099	rs638099	
rs11601808	rs11601808	
rs521893	rs521893	
rs627041	rs627041	
rs499945	rs499945	
rs590928	rs590928	
rs556609	rs556609	
rs687502	rs687502	
rs632100	rs632100	
rs2155043	rs2155043	
rs10895871	rs10895871	
rs10895872	rs10895872	
rs7932892	rs7932892	
rs7944207	rs7944207	
rs1938970	rs1938970	
rs1938971	rs1938971	
rs7928594	rs7928594	
rs2028816	rs2028816	
rs1938964	rs1938964	
rs639076	rs639076	
rs12290919	rs12290919	
rs1445622	rs1445622	

	rs11226835	rs11226835	
	rs519193		
	rs1938965		
	rs10895870		
	rs11226822		
	rs1037090		
	rs1445617		
	rs1822989		
**rs3138144**	rs3138144	rs3138144	rs3138144
RDH5	rs3138137	rs3138137	rs3138137
**rs2184971**	rs2184971	rs2184971	rs2184971
PCCA	rs2152881	rs2152881	rs2152881
	rs2390401	rs2390401	
**rs8000973**	rs8000973	rs8000973	rs8000974
ZIC2 & NDUFA12P1 (pseudogene)/	rs12018015	rs12018015	
ASNSP3 (pseudogene)	rs3848025		
	rs9517967		
	rs9517968		
	rs7321870		
	rs7320677		
	rs9517971		
	rs9517973		
	rs9517974		
	rs12870800		
	rs9513722		
	rs9513723		
	rs7322740		
**rs524952**	rs524952	rs524952	rs524953
LOC105370762/ GJD2	rs634990	rs634990	
	rs16959560		
	rs685352		
**rs4778879**	rs4778879	rs4778879	rs4778880
RASGRF1	rs7183818	rs7183818	
	rs6495366	rs6495366	
	rs6495367		
	rs755362		
	rs28412916		

	rs13380109	
	rs13380104	
	rs1007365	
	rs1007366	
	rs1961579	
	rs4778634	
	rs3851677	
	rs745030	
	rs4778882	
	rs745029	
**rs17183295**	rs17183295	rs17183295
MYO1D	rs62070164	rs62070164
	rs17780981	rs17780981
	rs17183600	rs17183600
	rs17183628	rs17183628
	rs17781136	rs17781136
	rs62070187	rs62070187
	rs75782470	rs75782470
	rs62068432	rs62068432
	rs1990825	rs1990825
	rs17183176	rs17183176
	rs4795719	rs4795719
	rs9898762	rs9898762
	rs75822124	rs75822124
	rs9303654	rs9303654
	rs9912761	rs9912761
	rs9911168	rs9911168
	rs75393526	rs75393526
	rs78418651	rs78418651
	rs17183113	rs17183113
	rs77819475	rs77819475
	rs17781142	rs17781142
	rs62070229	rs10512441
	rs62070232	
	rs10512441	
	rs62067160	
	rs62067167	
	rs80212824	
	rs17781005	
	rs113602044	
	rs62070183	

	rs76373015<br>rs79957065<br>rs79640811		
**rs4793501**<br>***KCNJ2 & CALM2P1 (pseudogene)/ LOC105371884***	rs4793501	rs4793501	rs4793501
**rs12971120**<br>***CNDP2***	rs12971120<br>rs3829640<br>rs2278162<br>rs2278161<br>rs734559<br>rs4891559<br>rs34676212<br>rs7241558<br>rs8084410<br>rs8084109<br>rs3764509<br>rs8083001<br>rs3794955<br>rs11151960<br>rs11151961<br>rs4891558<br>rs4891557<br>rs2278159<br>rs2278158<br>rs71359051<br>rs12968731<br>rs12608037<br>rs2303463<br>rs12605820<br>rs3794953<br>rs3764510<br>rs2241510<br>rs3794950<br>rs747176	rs12971120<br>rs3829640<br>rs2278162<br>rs2278161<br>rs734559<br>rs4891559<br>rs34676212<br>rs7241558<br>rs8084410<br>rs8084109<br>rs3764509<br>rs8083001	rs12971120<br>rs3829640<br>rs2278162<br>rs2278161<br>rs734559
**rs12193446**<br>***LAMA2***	rs12193446	rs12193446	rs12193446
**rs1381566**	rs1381566	rs1381566	rs1381566

LRRC4C	rs11602008		
	rs11606250		
**rs17648524**	rs17648524	rs17648524	rs17648524
RBFOX1	rs10500355	rs10500355	
	rs7184522	rs7184522	
	rs4332760	rs4332760	
**rs7744813**	rs7744813	rs7744813	rs7744813
KCNQ5			
**rs3138142**	rs3138142	rs3138142	rs3138142
RDH5	rs3138141	rs3138141	
**rs2137277**	rs2137277	rs2137277	rs2137277
ZMAT4/ SFRP1	rs2137278	rs2137278	rs2137278
	rs72644322	rs72644322	rs72644322
	rs4736886	rs4736886	
	rs35015019	rs35015019	
	rs4736884	rs4736884	
	rs72632903	rs72632903	
	rs7829127	rs7829127	
	rs7005382	rs7005382	
	rs6994354	rs16890057	
	rs16890057	rs869422	
	rs869422		
	rs16890054		
	rs11776583		
	rs72641600		
	rs11774708		
	rs17648449		
	rs13267110		
	rs11779358		
	rs7827879		
	rs11779532		
	rs11776113		
	rs11780608		
	rs9969525		
	rs6985757		
	rs11780139		
	rs35313163		
	rs35334145		
	rs11779864		

	rs35365471		
	rs13251462		
	rs6474280		
	rs6474283		
	rs7816573		
	rs7817426		
	rs7821235		
**rs1550094**	rs1550094	rs1550094	rs1550094
PRSS56	rs2117770		
	rs2741290		
	rs2141422		
	rs2741289		
	rs2853441		
**rs2908972**	rs2908972	rs2908972	rs2908972
SHISA6			
**rs17412774**	rs17412774	rs17412774	rs17412774
PABPCP2/ RNU7-2P (pseudogene)	rs10928276		
& PABPCP2	rs17491167		
	rs10803514		
	rs2130860		
	rs61051986		
	rs4662453		
	rs1399669		
	rs1399668		
	rs7604349		
	rs7577806		
	rs72854321		
	rs17491258		
	rs2035788		
	rs2035789		
	rs12611909		
	rs12621488		
	rs11675490		
	rs11681759		
	rs2201049		
	rs59126787		
	rs62168236		
	rs1515250		
	rs1515248		

	rs1515247		
	rs11691697		
	rs6717099		
	rs12612023		
	rs62169493		
	rs56075542		
	rs61049169		
	rs17733267		
	rs17789747		
	rs1515232		
	rs7602000		
	rs11674960		
**rs11145746**	rs11145746	rs11145746	rs11145746
TJP2	rs56207218	rs56207218	rs56207218
	rs11145657	rs11145657	rs11145657
	rs4515615	rs4515615	rs4515615
	rs11145588	rs11145588	rs11145588
	rs11145582	rs11145582	rs11145582
	rs7469887	rs7469887	rs7469887
	rs1538583	rs1538583	rs1538583
	rs11145488	rs11145488	rs11145488
	rs11145465	rs11145465	rs11145465
	rs11145442	rs11145442	rs11145442
	rs11145461	rs11145461	
	rs11145422	rs11145422	
	rs11145421	rs11145421	
	rs11145415	rs11145415	
	rs11145411	rs11145411	
	rs11145326		
	rs11145351		
**rs28412916**	rs28412916	rs28412916	rs28412916
RASGRF1	rs13380109	rs13380109	rs13380109
	rs13380104	rs13380104	rs13380104
	rs1007365	rs1007365	rs1007365
	rs1007366	rs1007366	rs1007366
	rs755362	rs755362	rs755362
	rs1961579	rs1961579	rs1961579
	rs4778634	rs4778634	rs4778634
	rs3851677	rs3851677	rs3851677
	rs6495367	rs6495367	rs6495367

	rs745030	rs745030	rs745030
	rs4778882	rs4778882	rs4778882
	rs745029	rs745029	
	rs1443658		
	rs1443657		
	rs2049176		
	rs6495366		
	rs7183818		
	rs4778879		
	rs2870111		
	rs12595749		
	rs4778918		
	rs1915724		
**rs5022942**	rs5022942	rs5022942	rs5022942
BMP3	rs4389567		
	rs12642476		
	rs985328		
	rs1495637		
	rs10007784		
**rs745480**	rs745480	rs745480	rs745480
LRIT2/ LRIT1& RGR	rs745479	rs745479	
	rs11200931	rs11200931	
	rs7909901	rs7909901	
	rs6585845	rs6585845	
	rs3814213	rs3814213	
	rs4933313	rs4933313	
	rs7092674	rs7092674	
	rs7089142		
	rs736006		
	rs4933985		
	rs6585847		
	rs4933314		
	rs7074150		
	rs4933980		
	rs4244947		
	rs4562752		
	rs4424615		
	rs10509491		
	rs1059341		
	rs7073076		

	rs11200922		
	rs4933976		
	rs4933975		
	rs10788333		
**rs2155413**	rs2155413	rs2155413	rs2155413
DLG2	rs1073986		
	rs10736767		
	rs2374576		
	rs10751116		
	rs4943906		
**rs13091182**	rs13091182	rs13091182	rs13091182
ZBTB38	rs35225290	rs35225290	rs35225290
	rs13066993	rs13066993	rs13066993
	rs13099193	rs13099193	rs13099193
	rs6785073	rs6785073	rs6785073
	rs6767899	rs6767899	rs6767899
	rs13069734	rs13069734	rs13069734
	rs6789653	rs6789653	rs6789653
	rs6767786	rs6767786	rs6767786
	rs9822327	rs9822327	rs9822327
	rs9822195	rs9822195	rs9822195
	rs9821337	rs9821337	rs9821337
	rs9858603	rs9858603	rs9858603
	rs13068065	rs13068065	rs13068065
	rs13067032	rs13067032	rs13067032
	rs2011092	rs2011092	
	rs6807935		
**rs17400325**	rs17400325	rs17400325	rs17400325
PDE11A & LOC105373764			
**rs17428076**	rs17428076	rs17428076	rs17428076
HAT1& LOC100420002 (pseudogene)/	rs72890842	rs72890842	
METAP1D & DLX1	rs62183762	rs62183762	
	rs62183765	rs62183765	
	rs55864623	rs55864623	
	rs72889094	rs72889094	
	rs56401580	rs56401580	
	rs17581439	rs17500114	
	rs62182374	rs17615262	

	rs62182373	rs3765166	
	rs62182372	rs3736499	
	rs72885532	rs12622895	
	rs72885529	rs3770455	
	rs17615262		
	rs17581361		
	rs17581347		
	rs12614709		
	rs12619041		
	rs56218609		
	rs62183808		
	rs6761111		
	rs17428174		
	rs55863868		
	rs72892835		
	rs17500114		
	rs62182430		
	rs72889848		
	rs57775770		
	rs12622895		
	rs12053046		
	rs3770455		
	rs57718990		
	rs6749895		
	rs3821093		
	rs3736499		
	rs55957712		
	rs6704590		
	rs62183852		
	rs62182392		
	rs3765166		
	rs788168		
	rs788164		
**rs6480859**	rs6480859	rs6480859	rs6480859
KCNMA1	rs11002118	rs11002118	rs11002118
	rs10824526	rs10824526	rs10824526
	rs11002117	rs11002117	rs11002117
	rs7919150	rs7919150	rs7919150
	rs7923107	rs7923107	rs7923107
	rs10824525	rs10824525	rs10824525
	rs10824523	rs10824523	rs10824523

	rs10509386	rs10509386	
	rs11002120	rs11002120	
	rs10824527	rs10824527	
	rs11002123	rs11002123	
	rs17486753	rs17486753	
	rs10509387	rs10509387	
	rs10824531	rs10824531	
	rs11002122	rs11002122	
	rs71475646	rs71475646	
	rs10509385		
	rs11002121		
	rs11002125		
	rs10824520		
	rs11002133		
	rs7918140		
	rs7903931		
	rs11002137		
	rs10824534		
	rs11002140		
	rs12358397		
	rs12355027		
	rs17407165		
	rs11002109		
	rs10824516		
	rs10824515		
	rs11002108		
	rs10824519		
	rs7897426		
	rs10824518		
	rs7895108		
	rs11002126		
	rs10824530		
**rs4291789**	rs4291789	rs4291789	rs4291789
ZIC2 & LINC00554/ NDUFA12P1 (pseudogene)	rs35266498	rs35266498	

*The nearest genes loci. One gene was listed when SNP located in gene, and two or more than two genes were listed when SNP located between genes (upstream/downstream).

Supplementary Table S2. Chromosome location and RegulomeDB scores for all index and proxy SNPs at r^2 threshold of 0.8.

Chromosome location	RefSNP	RegulomeDB Scores
chr17:31239644	rs10512441	1a
chr1:207472188	rs2802236	1b
chr1:207483444	rs2564974	1b
chr1:207494415	rs2564978	1b
chr9:71791546	rs1538583	1b
chr18:72168485	rs8084410	1b
chr1:207456473	rs12095015	1d
chr1:207502533	rs6700168	1d
chr12:56115584	**rs3138142**	1d
chr17:31184630	rs17183628	1d
chr18:72177231	rs11151960	1d
chr1:207387765	rs1346720	1f
chr1:207424726	rs7545125	1f
chr1:207470459	**rs1652333**	1f
chr1:207478979	rs1572275	1f
chr1:207501210	rs4844592	1f
chr1:207506328	rs10864231	1f
chr1:207507480	rs1507758	1f
chr1:207509364	rs1507760	1f
chr9:71733141	rs11145326	1f
chr9:71770938	rs11145488	1f
chr12:56114768	**rs3138144**	1f
chr17:31187215	rs17781142	1f
chr18:72154930	rs747176	1f
chr18:72167123	rs3764509	1f
chr18:72167801	rs12605820	1f
chr18:72168607	rs2303463	1f
chr18:72170298	rs4891557	1f
chr18:72170396	rs4891559	1f
chr18:72174022	**rs12971120**	1f
chr18:72174979	rs3829640	1f
chr18:72176082	rs2278161	1f
chr18:72178160	rs2278159	1f
chr18:72178299	rs2278158	1f
chr18:72179578	rs734559	1f
chr18:72182964	rs3794950	1f
chr10:60315642	rs1820542	2a
chr1:207489958	rs6671700	2b
chr1:207490318	rs891378	2b
chr1:207496998	rs2782828	2b
chr2:172680871	rs12614709	2b

chr2:172667894	rs3770455	2b
chr2:172878553	rs6704590	2b
chr2:172917798	rs788168	2b
chr3:53857802	rs12676	2b
chr3:141150989	rs6789653	2b
chr8:61631570	rs10106752	2b
chr9:71736687	rs11145351	2b
chr10:60269325	rs11006169	2b
chr10:60271530	rs7087998	2b
chr10:60271939	rs7095501	2b
chr10:60272247	rs7074778	2b
chr10:60276905	rs7098326	2b
chr10:60277488	rs1365741	2b
chr10:60290969	rs1427222	2b
chr10:60330421	rs1658429	2b
chr10:60276181	rs1896244	2b
chr15:79381704	rs745030	2b
chr15:79381704	rs745030	2b
chr15:79403584	rs2870111	2b
chr18:72180518	rs3794953	2b
chr1:207474364	rs77542432	3a
chr1:207476140	rs2802238	3a
chr1:207491488	rs891376	3a
chr1:207500292	rs4844591	3a
chr1:207501871	rs6700079	3a
chr1:207502346	rs6702997	3a
chr1:207508491	rs1354942	3a
chr1:207508729	rs7555030	3a
chr1:207414375	rs113231892	3a
chr1:207519521	rs112172464	3a
chr1:207519522	rs111911746	3a
chr2:233406997	**rs1881492**	3a
chr2:172527237	rs72889848	3a
chr2:172913389	rs788164	3a
chr3:141129998	rs13066993	3a
chr4:81924727	rs17005016	3a
chr4:81925095	rs963857	3a
chr8:61632195	rs13264416	3a
chr8:61664213	rs876957	3a
chr8:61665576	rs1473446	3a
chr8:61704054	rs13280978	3a
chr8:40757401	rs35334145	3a
chr9:71805628	rs4515615	3a
chr10:60268269	rs717452	3a

chr10:94938227	rs6583867	3a
chr10:94940766	rs11187313	3a
chr10:60269690	rs34774248	3a
chr10:60273897	rs10763551	3a
chr10:60276368	rs11006170	3a
chr10:60277054	rs7097576	3a
chr10:60291547	rs1593678	3a
chr10:60291641	rs1593677	3a
chr10:60276067	rs1896246	3a
chr10:60314182	rs1658446	3a
chr10:60330530	rs7921371	3a
chr10:60348885	rs10740731	3a
chr10:60276082	rs1896245	3a
chr10:79043416	rs11002108	3a
chr10:79052065	rs10824516	3a
chr11:105563858	rs10895860	3a
chr11:105605288	rs7932892	3a
chr12:56116980	rs3138137	3a
chr12:56115777	rs3138141	3a
chr17:31073640	rs75782470	3a
chr17:31142040	rs76373015	3a
chr18:72168311	rs8084109	3a
chr18:72177339	rs11151961	3a
chr18:72180738	rs2241510	3a
chr10:79068689	rs10824519	3b
chr1:207433348	rs10864175	4
chr1:207455420	rs2017760	4
chr1:207474396	rs2782845	4
chr1:207476039	rs971317	4
chr1:207482969	rs2802217	4
chr1:207502371	rs6703002	4
chr1:207502890	rs7548463	4
chr1:207503431	rs925130	4
chr1:207503720	rs925131	4
chr1:207506605	rs12133548	4
chr1:207506648	rs11120733	4
chr1:207507627	rs1507759	4
chr1:207509798	rs1507761	4
chr1:207510547	rs10746462	4
chr1:207510595	rs10746463	4
chr1:207510595	rs112720427	4
chr1:207519136	rs113896552	4
chr2:233387634	rs733603	4
chr2:233387634	rs733603	4

chr2:233408238	rs2573206	4
chr2:233408238	rs2573206	4
chr2:146882414	rs56075542	4
chr2:146888707	rs61049169	4
chr2:233385395	**rs1550094**	4
chr2:172759775	rs56401580	4
chr3:53837970	rs4687586	4
chr3:53847407	**rs14165**	4
chr3:141128803	rs13099193	4
chr3:141133959	**rs13091182**	4
chr3:141139329	rs6785073	4
chr4:81941455	rs6814223	4
chr4:81973898	rs985328	4
chr6:129834628	**rs12205363**	4
chr6:129820037	**rs12193446**	4
chr8:40741607	rs11779358	4
chr8:40757391	rs35313163	4
chr9:77149836	**rs7042950**	4
chr10:60271986	rs7073504	4
chr10:60272368	rs11817440	4
chr10:60273120	rs7100474	4
chr10:60273154	rs7079648	4
chr10:60273429	rs7078900	4
chr10:60273976	rs10763552	4
chr10:60274969	rs1427198	4
chr10:60275149	rs1427197	4
chr10:60276290	rs1896243	4
chr10:60277088	rs7098606	4
chr10:60277548	rs1365740	4
chr10:60278572	rs1427196	4
chr10:60288932	rs1658483	4
chr10:60289030	rs1649015	4
chr10:60290965	rs1427223	4
chr10:60295315	rs1658467	4
chr10:60297573	rs12259990	4
chr10:60274048	rs10763553	4
chr10:60278966	rs1427195	4
chr10:60291693	rs1593675	4
chr10:60313173	rs1658447	4
chr10:60314274	rs1620186	4
chr10:60327526	rs1658435	4
chr10:60330040	rs1649039	4
chr10:60330438	rs1649038	4
chr10:60276914	rs7098329	4

chr10:85960667	rs4933976	4
chr10:79051775	rs10824515	4
chr10:79057222	rs17407165	4
chr10:79061457	rs7895108	4
chr10:79065151	rs7897426	4
chr10:79080983	rs11002118	4
chr10:79084780	rs7919150	4
chr10:79099119	rs10509387	4
chr10:79099712	rs10824531	4
chr10:79111390	rs11002133	4
chr10:79137837	rs10824534	4
chr11:105541742	rs1445617	4
chr13:100728200	rs9517971	4
chr15:79373676	rs6495366	4
chr15:79381778	rs745029	4
chr15:79382018	rs4778882	4
chr15:79373676	rs6495366	4
chr15:79381778	rs745029	4
chr15:79382018	rs4778882	4
chr17:31132528	rs17781005	4
chr17:31221316	rs79640811	4
chr18:72166845	rs3764510	4
chr1:207413760	rs10864156	5
chr1:207419031	rs1864370	5
chr1:207420326	rs1429906	5
chr1:207422161	rs7533825	5
chr1:207442363	rs1367067	5
chr1:207448050	rs10864181	5
chr1:207483956	rs2564975	5
chr1:207486114	rs11120688	5
chr1:207504668	rs2184476	5
chr1:207504747	rs1507757	5
chr1:207508949	rs7512422	5
chr1:207521900	rs112511970	5
chr2:233379932	rs2853443	5
chr2:233379932	rs2853443	5
chr2:233379940	**rs1656404**	5
chr2:233379940	rs1656404	5
chr2:146759839	rs7602000	5
chr2:146801176	rs10803514	5
chr2:146813148	rs61051986	5
chr2:146860143	rs11681759	5
chr2:146860624	rs2201049	5
chr2:146889603	rs17733267	5

chr2:233374782	rs2853441	5
chr2:233374844	rs2741289	5
chr2:233375467	rs2741290	5
chr2:172684031	rs72885529	5
chr2:172684032	rs72885532	5
chr2:172886147	rs17428174	5
chr2:172888508	rs55863868	5
chr2:172896586	rs17500114	5
chr2:172693779	rs3765166	5
chr2:172694586	rs3821093	5
chr2:172875441	rs6749895	5
chr3:53847793	rs893362	5
chr3:141095371	rs13067032	5
chr3:141095723	rs13068065	5
chr3:141095948	rs9858603	5
chr3:141096735	rs9822195	5
chr3:141104179	rs6767786	5
chr3:141124606	rs2011092	5
chr3:141130834	rs35225290	5
chr3:141148418	rs13069734	5
chr4:81913992	rs11099457	5
chr4:81918620	rs2868079	5
chr4:81959965	**rs5022942**	5
chr4:81960464	rs4389567	5
chr6:73643288	**rs7744813**	5
chr8:40723037	rs16890054	5
chr8:40736662	rs72644322	5
chr8:40739691	rs4736884	5
chr8:40742056	rs72632903	5
chr8:40743751	rs4736886	5
chr8:60181300	rs7812962	5
chr8:61598553	rs4738814	5
chr8:61613024	rs4738816	5
chr8:61616842	rs35331770	5
chr8:61618712	rs1017861	5
chr8:61656510	rs7011801	5
chr8:61662347	rs28710955	5
chr8:61727586	rs4738828	5
chr8:61727908	rs4738829	5
chr8:40723037	rs16890054	5
chr8:40736662	rs72644322	5
chr8:40739691	rs4736884	5
chr8:40740935	rs13267110	5
chr8:40742056	rs72632903	5

chr8:40743751	rs4736886	5
chr8:40749373	rs11779532	5
chr8:40749959	rs11776113	5
chr8:40751023	rs11780608	5
chr8:40751703	rs9969525	5
chr8:40754420	rs11780139	5
chr8:40759407	rs11779864	5
chr8:40760900	rs35365471	5
chr8:40763734	rs13251462	5
chr8:40773798	rs7821235	5
chr9:77140950	rs10869412	5
chr9:71763361	rs11145442	5
chr9:71827134	rs56207218	5
chr10:60267136	rs1346302	5
chr10:60269737	rs36097682	5
chr10:60269754	rs1427201	5
chr10:60270234	rs7921207	5
chr10:60270259	rs7910442	5
chr10:60271371	rs7091440	5
chr10:60274181	rs10826198	5
chr10:60274212	rs10826199	5
chr10:60274605	rs7897298	5
chr10:60274927	rs1427200	5
chr10:60274940	rs1427199	5
chr10:60276828	rs7094882	5
chr10:60281089	rs1658493	5
chr10:60285819	rs1649023	5
chr10:60285866	rs1649022	5
chr10:60285923	rs1658489	5
chr10:60289338	rs1658481	5
chr10:60289373	rs1658480	5
chr10:60289388	rs1649092	5
chr10:60289397	rs1658479	5
chr10:60289439	rs1649091	5
chr10:60290619	rs1427224	5
chr10:60292433	rs1649082	5
chr10:60292443	rs1649081	5
chr10:60292663	rs1658468	5
chr10:60295341	rs1649074	5
chr10:60268397	rs717453	5
chr10:60278139	rs12264028	5
chr10:60278254	rs12248958	5
chr10:60304863	rs1649068	5
chr10:60305915	rs1649066	5

chr10:60306385	rs1649065	5
chr10:60306769	rs1658454	5
chr10:60308406	rs1614258	5
chr10:60310479	rs1649060	5
chr10:60311113	rs1658452	5
chr10:60311526	rs1658451	5
chr10:60316925	rs1658443	5
chr10:60318896	rs1658442	5
chr10:60320044	rs1649055	5
chr10:60326582	rs1658438	5
chr10:60327046	rs1649045	5
chr10:60327733	rs1621227	5
chr10:60328087	rs1626606	5
chr10:60328208	rs1649043	5
chr10:60329068	rs1658432	5
chr10:60333002	rs1658418	5
chr10:60333259	rs2393448	5
chr10:60339097	rs4948523	5
chr10:60341397	rs10763556	5
chr10:60341933	rs10763558	5
chr10:60352887	rs10740732	5
chr10:60288530	rs1649017	5
chr10:60288539	rs1649016	5
chr10:60288542	rs1658484	5
chr10:60306088	rs1658455	5
chr10:60321996	rs1649051	5
chr10:60330916	rs1658428	5
chr10:60333038	rs1658417	5
chr10:60341514	rs10763557	5
chr10:60365754	rs4245599	5
chr10:60266838	rs1863664	5
chr10:60267309	rs1346301	5
chr10:60267639	rs1427204	5
chr10:60267916	rs1427202	5
chr10:60268598	rs4113952	5
chr10:60268782	rs6481400	5
chr10:60268879	rs6481401	5
chr10:60268976	rs6481402	5
chr10:60269099	rs7917717	5
chr10:94926188	rs56871408	5
chr10:94927738	rs11187304	5
chr10:94937674	rs12357177	5
chr10:94942675	rs55744367	5
chr10:94949807	rs7920484	5

chr10:94953257	rs17382981	5
chr10:85960148	rs10788333	5
chr10:85960394	rs4933975	5
chr10:85961757	rs11200922	5
chr10:85962649	rs7073076	5
chr10:85974235	rs3814213	5
chr10:85976964	rs1059341	5
chr10:85980727	rs4933314	5
chr10:85981800	rs6585847	5
chr10:85986553	**rs745480**	5
chr10:85986695	rs745479	5
chr10:85989878	rs11200931	5
chr10:85991082	rs7909901	5
chr10:79052562	rs11002109	5
chr10:79066772	rs12355027	5
chr10:79070781	rs71475646	5
chr10:79077270	rs10824525	5
chr10:79079747	rs11002117	5
chr10:79081947	**rs6480859**	5
chr10:79086858	rs10509385	5
chr10:79090111	rs17486753	5
chr10:79095024	rs11002126	5
chr10:79114119	rs7918140	5
chr10:79124593	rs11002137	5
chr11:105563479	rs1838668	5
chr11:105564196	rs1445618	5
chr11:105571838	rs2028816	5
chr11:105576956	rs639076	5
chr11:105577199	rs638099	5
chr11:105583606	rs12290919	5
chr11:105585682	rs499945	5
chr11:105586570	rs590928	5
chr11:105589768	rs1938965	5
chr11:105610204	rs7928594	5
chr11:40149299	rs11606250	5
chr11:40149304	rs11602008	5
chr11:40149606	**rs1381566**	5
chr13:100724927	rs9517967	5
chr13:100728476	rs9517973	5
chr13:100728699	rs9517974	5
chr13:100736484	rs7322740	5
chr13:100672920	**rs4291789**	5
chr15:35006072	rs634990	5
chr15:35008334	rs685352	5

chr15:79372412	rs7183818	5
chr15:79372874	**rs4778879**	5
chr15:79376600	rs755362	5
chr15:79378166	**rs28412916**	5
chr15:79378774	rs13380109	5
chr15:79378820	rs13380104	5
chr15:79379491	rs1007365	5
chr15:79379540	rs1007366	5
chr15:79380515	rs1961579	5
chr15:79380821	rs4778634	5
chr15:79380868	rs3851677	5
chr15:79378166	rs28412916	5
chr15:79378774	rs13380109	5
chr15:79378820	rs13380104	5
chr15:79379491	rs1007365	5
chr15:79372412	rs7183818	5
chr15:79372874	rs4778879	5
chr15:79376600	rs755362	5
chr15:79379540	rs1007366	5
chr15:79380515	rs1961579	5
chr15:79380821	rs4778634	5
chr15:79380868	rs3851677	5
chr15:79386365	rs1443658	5
chr15:79386945	rs1443657	5
chr15:79392547	rs2049176	5
chr15:79434039	rs4778918	5
chr15:79441026	rs1915724	5
chr16:7459346	rs10500355	5
chr17:31029124	rs17183113	5
chr17:31035017	rs75393526	5
chr17:31036153	rs9911168	5
chr17:31036764	rs9303654	5
chr17:31078271	**rs17183295**	5
chr17:31103974	rs4795719	5
chr17:31119036	rs17780981	5
chr17:31142748	rs79957065	5
chr17:31190723	rs62070187	5
chr17:31251710	rs62067167	5
chr18:72169394	rs12608037	5
chr18:72170005	rs34676212	5
chr18:72170358	rs4891558	5
chr18:72174826	rs3794955	5
chr18:72175813	rs2278162	5
chr1:207422995	rs877049	6

chr1:207425042	rs7543250	6
chr1:207436814	rs4611012	6
chr1:207440850	rs6540896	6
chr1:207443552	rs6703857	6
chr1:207445760	rs10864180	6
chr1:207471223	rs2802233	6
chr1:207471588	rs2802234	6
chr1:207475595	rs971318	6
chr1:207488003	rs1858001	6
chr1:207488767	rs2914937	6
chr1:207526592	rs2135923	6
chr1:207513217	rs116218947	6
chr1:207516498	rs79515725	6
chr1:207526592	rs113849985	6
chr2:146759189	rs11674960	6
chr2:146762575	rs1515232	6
chr2:146798738	rs17491167	6
chr2:146806473	rs6717099	6
chr2:146810402	rs2130860	6
chr2:146817478	rs62168236	6
chr2:146820231	rs4662453	6
chr2:146822908	rs12612023	6
chr2:146826110	rs1399668	6
chr2:146830646	rs7577806	6
chr2:146831279	rs1515250	6
chr2:146831737	rs72854321	6
chr2:146835749	rs1515247	6
chr2:146836051	rs2035788	6
chr2:146837223	rs2035789	6
chr2:146838926	rs11691697	6
chr2:146839536	rs59126787	6
chr2:146850673	rs12611909	6
chr2:146876921	rs62169493	6
chr2:233375783	rs2117770	6
chr2:172600715	rs12619041	6
chr2:172682600	rs17581347	6
chr2:172682611	rs17581361	6
chr2:172683480	rs17615262	6
chr2:172684486	rs62182372	6
chr2:172685681	rs62182374	6
chr2:172737266	rs17581439	6
chr2:172748689	rs55864623	6
chr2:172772836	rs62182430	6
chr2:172794768	rs72889094	6

chr2:172843277	rs72890842	6
chr2:172862313	rs56218609	6
chr2:172883567	rs6761111	6
chr2:172587955	rs55957712	6
chr2:172673398	rs12053046	6
chr2:172686303	rs12622895	6
chr2:172721761	rs62182392	6
chr2:172926210	rs62183852	6
chr3:53849509	rs4687587	6
chr3:141096184	rs9821337	6
chr4:81897004	rs10004901	6
chr4:81930813	**rs1960445**	6
chr4:81894985	rs4693767	6
chr4:81966094	rs12642476	6
chr4:81975673	rs1495637	6
chr8:40722868	rs72641600	6
chr8:40723969	rs869422	6
chr8:40734661	**rs2137277**	6
chr8:60178040	rs7832404	6
chr8:61611419	rs28600309	6
chr8:61618947	rs1017862	6
chr8:61642326	rs10283352	6
chr8:61701056	**rs4237036**	6
chr8:61717136	rs4738826	6
chr8:61718464	rs28609390	6
chr8:40734661	rs2137277	6
chr8:40722868	rs72641600	6
chr8:40723969	rs869422	6
chr8:40733019	rs11774708	6
chr8:40747611	rs7827879	6
chr8:40769229	rs6474283	6
chr8:40773507	rs7817426	6
chr9:77144042	rs7024262	6
chr9:77147708	rs13289812	6
chr9:77147719	rs13283886	6
chr9:77152626	rs10869416	6
chr9:77155381	rs10869418	6
chr9:71754152	rs11145415	6
chr9:71755704	rs11145422	6
chr9:71766118	rs11145461	6
chr9:71766592	rs11145465	6
chr9:71794380	rs7469887	6
chr9:71797443	rs11145582	6
chr9:71799195	rs11145588	6

chr9:71834379	**rs11145746**	6
chr10:60266554	rs12257983	6
chr10:60266639	rs1863665	6
chr10:94912648	rs10882157	6
chr10:94914770	rs11187298	6
chr10:94917412	rs12358810	6
chr10:94928178	rs117531235	6
chr10:94936622	rs7078201	6
chr10:94936643	rs7078310	6
chr10:94939601	rs10882169	6
chr10:94941926	rs7916761	6
chr10:94943012	rs4918865	6
chr10:94943889	rs10882172	6
chr10:94946267	rs7903804	6
chr10:94947317	rs12360392	6
chr10:94954807	rs72815184	6
chr10:94956240	rs72815186	6
chr10:94963390	rs72815193	6
chr10:60278339	rs12242287	6
chr10:60279323	rs1649034	6
chr10:60279633	rs1649033	6
chr10:60279829	rs1649030	6
chr10:60279908	rs1658496	6
chr10:60280322	rs1658494	6
chr10:60280427	rs1649028	6
chr10:60280910	rs1649027	6
chr10:60281583	rs1649025	6
chr10:60285477	rs28426375	6
chr10:60285493	rs3001714	6
chr10:60286158	rs2577393	6
chr10:60288080	rs1658486	6
chr10:60288110	rs1649019	6
chr10:60288149	rs1649018	6
chr10:60288330	rs1658485	6
chr10:60289550	rs1658478	6
chr10:60291907	rs1593674	6
chr10:60292382	rs1658470	6
chr10:60293293	rs1649080	6
chr10:60294378	rs1593672	6
chr10:60294684	rs1626532	6
chr10:60297101	rs1582827	6
chr10:60297661	rs12261597	6
chr10:60298940	rs1303968	6
chr10:60281266	rs1658492	6

chr10:60289694	rs1649089	6
chr10:60291910	rs1658471	6
chr10:60294624	rs1579394	6
chr10:60301293	rs7087528	6
chr10:60302514	rs1619284	6
chr10:60312710	rs12569559	6
chr10:60314728	rs2114564	6
chr10:60314814	rs2438182	6
chr10:60316210	rs7099881	6
chr10:60320445	rs1623568	6
chr10:60320663	rs1621811	6
chr10:60323932	rs1658440	6
chr10:60324094	rs1649049	6
chr10:60324281	rs1649048	6
chr10:60325985	rs1658439	6
chr10:60326108	rs1649046	6
chr10:60326909	rs1658436	6
chr10:60328755	rs1658434	6
chr10:60329124	rs3001717	6
chr10:60329229	rs2842083	6
chr10:60331503	rs1658427	6
chr10:60331927	rs1658421	6
chr10:60332700	rs1649035	6
chr10:60343029	rs2393449	6
chr10:60343526	rs12263522	6
chr10:60347409	rs10763559	6
chr10:60360833	rs893370	6
chr10:60279741	rs1649031	6
chr10:60285508	rs3001715	6
chr10:60293318	rs1649079	6
chr10:60293319	rs1649078	6
chr10:60295968	rs1593671	6
chr10:60300765	rs1593670	6
chr10:60305239	rs1649067	6
chr10:60308557	rs7914888	6
chr10:60309601	rs1658453	6
chr10:60323020	rs1658441	6
chr10:60327297	rs1649044	6
chr10:85966872	rs7092674	6
chr10:85975245	rs6585845	6
chr10:85977174	rs10509491	6
chr10:85978938	rs4244947	6
chr10:79083870	rs10824526	6
chr10:79085682	rs7923107	6

chr10:79087618	rs11002121	6
chr10:79088003	rs10824527	6
chr10:79097665	rs10824530	6
chr10:79114689	rs7903931	6
chr10:79141035	rs11002140	6
chr11:105553562	rs10895856	6
chr11:105561599	rs11226825	6
chr11:105565465	rs994575	6
chr11:105566241	rs61900312	6
chr11:105569355	rs10791767	6
chr11:105582612	rs519193	6
chr11:105587364	rs687502	6
chr11:105593542	rs7931588	6
chr11:105596418	rs1938963	6
chr11:105597152	rs2155043	6
chr11:105602879	rs10895871	6
chr11:105604718	rs10895872	6
chr11:105605117	rs11226835	6
chr11:105605818	rs7944207	6
chr11:105607715	rs1938971	6
chr11:84634789	**rs2155413**	6
chr11:84636899	rs10751116	6
chr11:84637064	rs10736767	6
chr11:84638734	rs1073986	6
chr11:84638825	rs4943906	6
chr13:100688670	rs9585326	6
chr13:100727589	rs7321870	6
chr13:100727677	rs7320677	6
chr13:100818091	**rs2184971**	6
chr13:100735420	rs9513723	6
chr15:35006599	rs16959560	6
chr16:7460698	rs7184522	6
chr17:31018702	rs113602044	6
chr17:31026840	rs77819475	6
chr17:31029547	rs78418651	6
chr17:31036492	rs9912761	6
chr17:31037893	rs75822124	6
chr17:31038658	rs9898762	6
chr17:31056291	rs1990825	6
chr17:31086432	rs62070164	6
chr17:31175078	rs17183600	6
chr17:31185041	rs17781136	6
chr17:31230092	rs62070232	6
chr17:31246584	rs62067160	6

chr17:68718733	**rs4793501**	6
chr18:72165057	rs8083001	6
chr18:72169266	rs7241558	6
chr18:72169707	rs12968731	6
chr18:72169722	rs71359051	6
chr1:207410964	rs7522701	No Data
chr1:207415562	rs6664815	No Data
chr1:207420858	rs6540888	No Data
chr1:207421571	rs10864167	No Data
chr1:207426564	rs1835307	No Data
chr1:207435828	rs1579484	No Data
chr1:207441496	rs11120586	No Data
chr1:207443570	rs6703860	No Data
chr1:207444927	rs59036171	No Data
chr1:207445677	rs10864178	No Data
chr1:207498643	rs6681853	No Data
chr1:207505708	rs6662070	No Data
chr1:207516360	rs113742197	No Data
chr2:146773947	**rs17412774**	No Data
chr2:146785402	rs10928276	No Data
chr2:146824807	rs1399669	No Data
chr2:146830540	rs7604349	No Data
chr2:146835247	rs17491258	No Data
chr2:146835661	rs1515248	No Data
chr2:146850813	rs12621488	No Data
chr2:146855991	rs11675490	No Data
chr2:146889664	rs17789747	No Data
chr2:233374982	rs2141422	No Data
chr2:172662613	rs57718990	No Data
chr2:172687730	rs57775770	No Data
chr2:172690417	rs3736499	No Data
chr2:172685554	rs62182373	No Data
chr2:172828797	rs62183762	No Data
chr2:172832023	rs62183765	No Data
chr2:172851935	**rs17428076**	No Data
chr2:172880074	rs62183808	No Data
chr2:172894613	rs72892835	No Data
chr2:178565912	**rs17400325**	No Data
chr3:53848671	rs920252	No Data
chr3:141096754	rs9822327	No Data
chr3:141114292	rs6807935	No Data
chr3:141125438	rs6767899	No Data
chr4:81923676	rs10003846	No Data
chr4:81892036	rs10049776	No Data

chr4:81906968	rs4693773	No Data
chr4:81925566	rs9307776	No Data
chr4:81927205	rs4458448	No Data
chr4:81977689	rs10007784	No Data
chr8:40710730	rs11776583	No Data
chr8:40726393	**rs7829127**	No Data
chr8:40726581	rs16890057	No Data
chr8:40729497	rs6994354	No Data
chr8:40734705	rs2137278	No Data
chr8:40738800	rs35015019	No Data
chr8:40752242	rs7005382	No Data
chr8:60179085	**rs7837791**	No Data
chr8:61626086	rs10104525	No Data
chr8:61667897	rs1483208	No Data
chr8:61695704	rs35168272	No Data
chr8:40710730	rs11776583	No Data
chr8:40726393	rs7829127	No Data
chr8:40726581	rs16890057	No Data
chr8:40729497	rs6994354	No Data
chr8:40734705	rs2137278	No Data
chr8:40738800	rs35015019	No Data
chr8:40739277	rs17648449	No Data
chr8:40752215	rs6985757	No Data
chr8:40752242	rs7005382	No Data
chr8:40766300	rs6474280	No Data
chr8:40772985	rs7816573	No Data
chr9:77144199	rs7867494	No Data
chr9:77154450	rs10869417	No Data
chr9:71753548	rs11145411	No Data
chr9:71754872	rs11145421	No Data
chr9:71815903	rs11145657	No Data
chr9:76348529	rs117588997	No Data
chr10:60265403	**rs7084402**	No Data
chr10:60265956	rs12250013	No Data
chr10:60266151	rs12259315	No Data
chr10:94916761	rs10882163	No Data
chr10:94924323	**rs10882165**	No Data
chr10:94940824	rs11187314	No Data
chr10:94941812	rs7916838	No Data
chr10:94941978	rs7917125	No Data
chr10:94945556	rs57346439	No Data
chr10:94946122	rs7919934	No Data
chr10:94950712	rs17108260	No Data
chr10:94962713	rs7894625	No Data

chr10:60277248	rs2170779	No Data
chr10:60278486	rs11006171	No Data
chr10:60279700	rs1649032	No Data
chr10:60280152	rs1658495	No Data
chr10:60281353	rs1658491	No Data
chr10:60281399	rs1649026	No Data
chr10:60281615	rs1658490	No Data
chr10:60286070	rs6481404	No Data
chr10:60289606	rs1658477	No Data
chr10:60292041	rs1649085	No Data
chr10:60292116	rs1649084	No Data
chr10:60292401	rs1658469	No Data
chr10:60293905	rs1649077	No Data
chr10:60294060	rs1649076	No Data
chr10:60298111	rs1649072	No Data
chr10:60298207	rs1658463	No Data
chr10:60298283	rs1658462	No Data
chr10:60298476	rs1303970	No Data
chr10:60299150	rs1658460	No Data
chr10:60282077	rs1632584	No Data
chr10:60286027	rs1649021	No Data
chr10:60294169	rs1593673	No Data
chr10:60299245	rs1658459	No Data
chr10:60301381	rs1658458	No Data
chr10:60302715	rs1658457	No Data
chr10:60303694	rs1649069	No Data
chr10:60304325	rs1658456	No Data
chr10:60309214	rs1649063	No Data
chr10:60309325	rs1649062	No Data
chr10:60312598	rs1649059	No Data
chr10:60317086	rs1649058	No Data
chr10:60318113	rs1649057	No Data
chr10:60327829	rs1620404	No Data
chr10:60328662	rs1649042	No Data
chr10:60328781	rs1649041	No Data
chr10:60328890	rs11815274	No Data
chr10:60329165	rs2917949	No Data
chr10:60329182	rs2577392	No Data
chr10:60331134	rs1649037	No Data
chr10:60331546	rs1658425	No Data
chr10:60331587	rs1658424	No Data
chr10:60331626	rs1658423	No Data
chr10:60331757	rs1658422	No Data
chr10:60331877	rs1649036	No Data

chr10:60332811	rs1658419	No Data
chr10:60338752	rs4141671	No Data
chr10:60343084	rs7474570	No Data
chr10:60343637	rs11006192	No Data
chr10:60351223	rs4948524	No Data
chr10:60359090	rs10826202	No Data
chr10:60360422	rs10763564	No Data
chr10:60264740	rs2033149	No Data
chr10:60265241	rs7099496	No Data
chr10:60287634	rs1346300	No Data
chr10:60297174	rs1582826	No Data
chr10:60318275	rs1158212	No Data
chr10:60331541	rs1658426	No Data
chr10:60361269	rs4948525	No Data
chr10:85966935	rs7089142	No Data
chr10:85971108	rs4933313	No Data
chr10:85978575	rs4424615	No Data
chr10:85978808	rs4562752	No Data
chr10:85979231	rs4933980	No Data
chr10:85980314	rs7074150	No Data
chr10:85985576	rs4933985	No Data
chr10:85985919	rs736006	No Data
chr10:79063541	rs10824518	No Data
chr10:79067052	rs12358397	No Data
chr10:79068963	rs10824520	No Data
chr10:79072452	rs10824523	No Data
chr10:79086957	rs10509386	No Data
chr10:79087030	rs11002120	No Data
chr10:79088889	rs11002122	No Data
chr10:79088921	rs11002123	No Data
chr10:79091400	rs11002125	No Data
chr11:105536017	rs1822989	No Data
chr11:105549734	rs1037090	No Data
chr11:105550937	rs11226822	No Data
chr11:105554202	rs10895857	No Data
chr11:105556006	rs1445607	No Data
chr11:105556597	**rs11601239**	No Data
chr11:105559724	rs7106983	No Data
chr11:105561553	rs977516	No Data
chr11:105565950	rs12790329	No Data
chr11:105566601	rs1822993	No Data
chr11:105568368	rs11226827	No Data
chr11:105568410	rs11226828	No Data
chr11:105570438	rs1445619	No Data

chr11:105572625	rs10895864	No Data
chr11:105574054	rs1373926	No Data
chr11:105578907	rs11601808	No Data
chr11:105581199	rs12417624	No Data
chr11:105582887	rs521893	No Data
chr11:105585342	rs627041	No Data
chr11:105586341	rs1445622	No Data
chr11:105587275	rs556609	No Data
chr11:105588856	rs632100	No Data
chr11:105591416	rs7926520	No Data
chr11:105596275	rs1938964	No Data
chr11:105596773	rs1954762	No Data
chr11:105601178	rs10895870	No Data
chr11:105605902	rs1938970	No Data
chr11:84638164	rs2374576	No Data
chr13:100691366	**rs8000973**	No Data
chr13:100724657	rs3848025	No Data
chr13:100726694	rs9517968	No Data
chr13:100805796	rs2390401	No Data
chr13:100817994	rs2152881	No Data
chr13:100730078	rs12870800	No Data
chr13:100735401	rs9513722	No Data
chr13:100705064	rs35266498	No Data
chr15:35005885	**rs524952**	No Data
chr15:79375346	rs6495367	No Data
chr15:79375346	rs6495367	No Data
chr15:79432358	rs12595749	No Data
chr16:7459682	**rs17648524**	No Data
chr16:7462044	rs4332760	No Data
chr17:31030457	rs80212824	No Data
chr17:31054124	rs17183176	No Data
chr17:31066496	rs62068432	No Data
chr17:31164366	rs62070183	No Data
chr17:31227592	rs62070229	No Data
chr17:11407258	**rs2908972**	No Data
